# Supplementary material for: What is the feasibility and patient acceptability of a digital system for arm and hand rehabilitation after stroke? A mixed-methods, single-arm feasibility study of the ‘OnTrack’ intervention for hospital and home use
Source: BMJ Open. 2022 Sep 28;12(9):e062042. doi: 10.1136/bmjopen-2022-062042 (PMC9528675; doi:10.1136/bmjopen-2022-062042)
Supplement: Supplementary data [file bmjopen-2022-062042supp001.pdf]

## Supplementary file 1 - Phase 1 and Phase 2 coaching structures

### Coaching delivery - Study phase 1

| Week | Session | Coaching phase | Coaching guidelines |
|---|---|---|---|
| W1 | Baseline assessment | *No coaching* | |
| W2 | Coaching session 1 | Onboarding | Introduction to OnTrack / purpose, value, practical issues |
| W3 | Coaching session 2 | Problem solving | Encouraging people to discuss areas where they struggle, then facilitate coming up with ideas and strategies around problems faced during daily life situations. |
| W4 | | | |
| W5 | Coaching session 3 | Self-discovery | Finding out new ways of doing things, trying out different activities, so that any attempt to try and find a new way around something is seen as a positive. |
| W6 | | | |
| W7 | Coaching session 4 | Goal setting | Working with participants to identify, plan and execute steps towards a goal regardless of size and ambition. The aim is to help participants learn and get comfortable with this process under their terms.<br><br>Outcome measure collection. |
| W8 | Halfway assessment | | |
| W9 | | | |
| W10 | Coaching session 5 | Reflection | Looking back at achievements and failures to understand what worked and what didn't in order to adjust if necessary and look forward to where they want to be. |
| W11 | | | |
| W12 | Coaching session 6 | Signposting | Using goals and aspirations to help participants create links that encourage social participation, community integration and/or peer support. |
| W13 | | | |
| W14 | Final assessment | *No coaching* | |

### Coaching delivery - Study phase 2

| Week | Session | Coaching phase | Coaching guidelines |
|---|---|---|---|
| W1 | Baseline assessment | *No coaching* | Initial contact; conversation about participant's background and present situation. |
| W2 | Coaching session 1 | Introduction | Focus on creating a habit of using OnTrack and charging devices. Setting the scene for coaching. |
| W3 | Coaching session 2 | OnTrack coaching | In-depth conversations about participants' aspirations and what's important to them.<br><br>Discussion to assess participants' understanding of stroke and rehab mechanisms, identify topics of curiosity, signpost as necessary.<br><br>Use of self-management principles (Problem Solving, Self- |
| W4 | | | |
| W5 | Coaching session 3 | | |

| | | | |
|---|---|---|---|
| W6 | | | discovery, Goal Setting, and Reflection) and rehab guidelines to focus conversations towards reaching an agreed activity plan (however small).<br><br>Make connections between activity performed and data recorded. |
| W7 | Halfway assessment | | |
| W8 | | | |
| W9 | Coaching session 4 | | |
| W10 | | | |
| W11 | Coaching session 5 | | |
| W12 | | | |
| W13 | Coaching session 6 | Signposting | Signpost to relevant resources that can be accessed by participants going forward. |
| W14 | Final assessment | *No coaching* | Closure; last query to ensure participants feel confident to continue their rehab on their own. |
