# Supplementary material for: What is the feasibility and patient acceptability of a digital system for arm and hand rehabilitation after stroke? A mixed-methods, single-arm feasibility study of the ‘OnTrack’ intervention for hospital and home use
Source: BMJ Open. 2022 Sep 28;12(9):e062042. doi: 10.1136/bmjopen-2022-062042 (PMC9528675; doi:10.1136/bmjopen-2022-062042)
Supplement: Author's manuscript [file bmjopen-2022-062042.draft_revisions.pdf]

BMJ Open is committed to open peer review. As part of this commitment we make the peer review history of every article we publish publicly available.

When an article is published we post the peer reviewers' comments and the authors' responses online. We also post the versions of the paper that were used during peer review. These are the versions that the peer review comments apply to.

The versions of the paper that follow are the versions that were submitted during the peer review process. They are not the versions of record or the final published versions. They should not be cited or distributed as the published version of this manuscript.

BMJ Open is an open access journal and the full, final, typeset and author-corrected version of record of the manuscript is available on our site with no access controls, subscription charges or pay-per-view fees (http://bmjopen.bmj.com).

If you have any questions on BMJ Open's open peer review process please email info.bmjopen@bmj.com

BMJ Open

Feasibility study of OnTrack: a digital system for arm and hand rehabilitation after stroke.

Journal:	*BMJ Open*
Manuscript ID	bmjopen-2022-062042
Article Type:	Original research
Date Submitted by the Author:	15-Feb-2022
Complete List of Authors:	Fusari, Gianpaolo; Imperial College London, Helix Centre<br>Gibbs, Ella; Imperial College London, Helix Centre<br>Hoskin, Lily; Imperial College London, Helix Centre<br>Lawrence-Jones, Anna; Imperial College London, Helix Centre<br>Dickens, Daniel; Imperial College London, Helix Centre<br>Fernandez Crespo, Roberto; Imperial College London<br>Leis, Melanie; Imperial College London, Big Data and Analytical Unit, Institute of Global Health Innovation<br>Crow, Jennifer; Imperial College Healthcare NHS Trust, Hyperacute Stroke Unit<br>Taylor, Elizabeth; Kingston University and St George's University of London<br>Jones, Fiona; Kingston University and St George's University of London<br>Darzi, Ara; Imperial College London, Institute of Global Health Innovation
Keywords:	Stroke < NEUROLOGY, REHABILITATION MEDICINE, NEUROLOGY

SCHOLARONE™
Manuscripts

I, the Submitting Author has the right to grant and does grant on behalf of all authors of the Work (as defined in the below author licence), an exclusive licence and/or a non-exclusive licence for contributions from authors who are: i) UK Crown employees; ii) where BMJ has agreed a CC-BY licence shall apply, and/or iii) in accordance with the terms applicable for US Federal Government officers or employees acting as part of their official duties; on a worldwide, perpetual, irrevocable, royalty-free basis to BMJ Publishing Group Ltd ("BMJ") its licensees and where the relevant Journal is co-owned by BMJ to the co-owners of the Journal, to publish the Work in this journal and any other BMJ products and to exploit all rights, as set out in our licence.

The Submitting Author accepts and understands that any supply made under these terms is made by BMJ to the Submitting Author unless you are acting as an employee on behalf of your employer or a postgraduate student of an affiliated institution which is paying any applicable article publishing charge ("APC") for Open Access articles. Where the Submitting Author wishes to make the Work available on an Open Access basis (and intends to pay the relevant APC), the terms of reuse of such Open Access shall be governed by a Creative Commons licence – details of these licences and which Creative Commons licence will apply to this Work are set out in our licence referred to above.

Other than as permitted in any relevant BMJ Author's Self Archiving Policies, I confirm this Work has not been accepted for publication elsewhere, is not being considered for publication elsewhere and does not duplicate material already published. I confirm all authors consent to publication of this Work and authorise the granting of this licence.

For peer review only

TITLE

Feasibility study of OnTrack: a digital system for arm and hand rehabilitation after stroke.

AUTHORS AND AFFILIATIONS

Gianpaolo Fusari[1], Ella Gibbs[1], Lily Hoskin[1], Anna Lawrence-Jones[1], Daniel Dickens[1], Roberto Fernandez Crespo[2], Melanie Leis[2], Jennifer Crow[3], Elizabeth Taylor[4], Fiona Jones[4], Ara Darzi[1]

[1]Helix Centre, Imperial College London and the Royal College of Art, London, United Kingdom

[2]Big Data and Analytical Unit, Institute of Global Health Innovation, Imperial College London, London, United Kingdom

[3]Stroke Unit, Charing Cross Hospital, Imperial College Healthcare NHS Trust, United Kingdom

[4]Kingston University and St George's, University of London, London, United Kingdom

Correspondence to:

Gianpaolo Fusari, Helix Centre, Imperial College London and the Royal College of Art, London, United Kingdom - gianpaolo@helixcentre.com

Word count:

3974

KEY WORDS

Stroke, rehabilitation medicine, neurology, public health, digital health

ABSTRACT

Objectives

Arm weakness is common after stroke; repetitive activity is critical for recovery but people struggle with knowing what to do, volume, and monitoring progress. We studied the feasibility and acceptability of OnTrack, a digital intervention supporting arm and hand rehabilitation in acute and community settings.

Design

A mixed method, single-arm study evaluating the feasibility of OnTrack for hospital and community use. An independent process evaluation assessed the intervention's fidelity, dose and reach. Amendments to the protocol were necessary after Covid-19.

Setting

Acute and community-based stroke services in North West London.

For peer review only

Participants

12 adults with a stroke diagnosis requiring arm and hand rehabilitation.

Intervention

12 weeks using the OnTrack system comprising arm tracking and coaching support for self-management.

Primary and secondary outcome measures

Recruitment, retention and completion rates; compliance and adherence to the intervention; reasons for study decline/withdrawal.

Intervention fidelity and acceptability, evaluated through an independent process evaluation.

Patient measures including activity baseline, healthcare activation, arm function & impairment collected at baseline, week 7 and week 14 of participation.

Results

181 individuals screened, 24 recruited, 12 completed the intervention. Recruitment, retention and completion were in line with pre-study expectations and acceptable for a definitive trial. Participants felt the study requirements were acceptable and the intervention usable. Fidelity of delivery was acceptable according to predetermined fidelity markers. Sample size estimates and primary outcomes for an RCT were identified.

Conclusions

The intervention was found to be usable and acceptable by participants; objectives were met and demonstrated that a definitive RCT would be feasible and acceptable.

Trial registration number

NCT03944486.

For peer review only

STRENGTHS AND LIMITATIONS

- This was the first feasibility trial of a novel intervention employing an integrated approach for tracking arm activity and self-management coaching with the aim of increasing the opportunities for independent rehabilitation.
- Recruitment for the study began in September 2019, modifications to the protocol were necessary to enable the delivery of the intervention remotely after the start of the Covid 19 pandemic in March 2020; the feasibility of the intervention was not affected.
- A Patient and Public Involvement (PPI) group met four times during the study. The group gave advice regarding modifications to the protocol, and contributed to the interpretation and dissemination of data findings.

For peer review only - http://bmjopen.bmj.com/site/about/guidelines.xhtml

- An independent process evaluation has provided detailed information about implementation, context, and the mechanisms of impact of the intervention. Findings have provided confirmation of intervention fidelity and training needs required for a definitive trial.
- For pragmatic reasons the study used a non-randomised design carried out at a single site; as such, it was not possible to determine whether the observed outcomes were due to the intervention, or a product of natural recovery.
- Longer term follow-up of participants was not possible within the timeframe set for the trial; however participant views were sought regarding the acceptability of longer-term follow-up.

INTRODUCTION

Background

Globally, five million people are left disabled from stroke, commonly with a form of arm impairment.(1) In the UK, 75% of disabled stroke survivors, or ~450,000 people, have an arm weakness.(2) By 2035, this number may increase by a third.(3) Stroke costs the UK society £26b annually, by 2025 costs could increase to £43b and to £75b by 2035. An ageing population, better stroke survival rates, and the overall increase in labour costs account for this trend.(3)

Understanding when to provide interventions that can improve arm function is recognised as a national research priority;(4) despite this, time spent providing therapy for the arm is often limited, resulting in patients spending minimal time rehabilitating or being active.(5,6) There is a correlation between physical activity and the ability to perform activities of daily living using the arm,(7) but a Cochrane review of over 500 trials failed to yield high-quality practice recommendations for arm interventions.(8) The Covid-19 pandemic exacerbated this problem resulting in thousands of stroke survivors receiving diminished rehabilitation and an increase in health inequalities.(9,10) Without specialist support, rehabilitation can be less effective and an isolating experience.(11)

Ethnographic studies describe how patients struggle to see and keep track of improvements(12-14) - especially outside of scheduled one-to-one therapy - having an impact on motivation and creating dependency on therapists for feedback.(6,7,15,16) Stroke survivors often report feeling unsupported after leaving hospital and not knowing how to best help themselves improve their arm function.

We believe repetitive activity can be increased by targeting the time patients go about their daily activities and could use their arm movement (however small) to a greater extent. Capacity for activity could be increased further by using self-management strategies as demonstrated by several programmes in stroke and other long-term conditions.(17-20) The aim of this study was to assess the feasibility of the OnTrack intervention - which could increase opportunities for arm activity by improving individuals' self-management skills through tailored support and real-time activity feedback - and inform the design of a definitive randomised controlled trial (RCT).

As per study protocol,(21) recruitment, adherence and retention rates were measured, as well as reasons for declining participation or withdrawing from the trial; the utility and completion of outcome measures to provide an indication of effectiveness to inform sample size calculations for an RCT; the usability of OnTrack by study participants; and the acceptability of study procedures by front-line staff. An independent process evaluation assessed the fidelity of the intervention delivery as well as the acceptability of the intervention to participants and delivery team.

For peer review only - http://bmjopen.bmj.com/site/about/guidelines.xhtml

For peer review only

METHODS

Design

A feasibility study with a nested process evaluation. The study was a single-site, non-randomised intervention trial. The design of the study was developed through a collaborative approach between the authors, a PPI steering group, stroke therapists (occupational therapists and physiotherapists) and the Research Design Service at the National Institute for Health Research (NIHR).

Study settings

Prior to lockdown restrictions caused by the Covid-19 pandemic, participants were enrolled in the study at an inner city NHS hospital Trust in West London and, where necessary, continued their participation in the community after discharge to complete the 14-week intervention period. After restrictions were introduced, all participants were enrolled at home and were followed remotely for 14 weeks.

Recruitment

Participants were recruited from the Hyperacute Stroke Unit (HASU), Acute Stroke Unit (ASU) and Clinical Neuro Rehabilitation Unit (CNRU) at an NHS Hospital Trust in London.

Recruitment followed two distinct procedures before and after lockdown restrictions were in place. Between August 2019 and March 2020, hospital therapy teams were responsible for screening, introducing the study, and providing information documents to potential participants. After taking consent, therapists shared patient information with the research team. Recruitment was suspended between March and August 2020. From September 2020 onwards, therapy teams continued to perform eligibility screens sharing patient information with the research team only at discharge. The consent and recruitment process was then completed in the community.

Intervention

The intervention was the OnTrack system, consisting of three main components:

1. Activity tracking. Participants wore a smartwatch on their affected arm which included software and a purpose-built algorithm providing data on gross arm activity (minutes of activity performed).

2. Motivational content. Delivered as visual feedback on the amount of activity performed, personalised in-app messages, and links to educational material. A sample of content can be seen in Table 1.

3. Self-management coaching. Delivered during 1-1 coaching sessions (in person and remotely). The coaching component has been influenced by the self-management principles as defined by the Bridges Self-Management Programme (22) and the Taking Charge After Stroke (23) self-management programme.

Activity data collected by participants was monitored remotely by the research team, this helped to guide conversations during coaching sessions.

The coaching component was reviewed at the study's halfway point following data analysis performed by the process evaluation team in discussion with the PPI group.

For peer review only - http://bmjopen.bmj.com/site/about/guidelines.xhtml

Participants were loaned all equipment necessary for the trial and no previous experience with using smart devices was required to participate. Technical support was provided by the intervention team in cases where the hardware and/or software failed to perform the required functions to deliver the intervention.

Table 1 - In-app messages

Messages and links to content related to stroke rehabilitation and self-management were sent to participants via the OnTrack smartphone application. Messages were divided into four different categories.

Message category	Description	Frequency, time of day	Sample message
Intention settings	Message to set the participant's intentions for the day. Participants were able to respond to these messages with a Yes / No answer.	Weekdays, in the morning	"Good morning Jon!<br><br>Aim to wear the watch on your LEFT wrist for as long as you can today and keep an eye on your arm activity target of 45 minutes.<br><br>Are you ready to try this?"
Tips & advice	Personalised messages to include tips or advice relevant to the participant's situation. This type of message did not require a response.	2-3 times per week, at midday	"Weekends are often different from the rest of the week but you can still include activities that involve your LEFT arm.<br><br>It's also ok to have a rest but don't forget to use appropriate cutlery for every meal if you have the opportunity as we discussed before.<br><br>You've got this!"
Reflective practice	Message with the intention to help participants reflect on their progress. This type of message did not require a response.	2-3 times per week, towards the end of the day	"Hi Jon!<br><br>I hope you managed to reach your target of 45 minutes of arm activity today.<br><br>Were you able to think about new ways of involving your arm?<br><br>Let's talk about these when we meet!"
Links to external content	Participants in Phase 1 of the study received a total of 9 links whilst participants in Phase 2 received 11 links signposting to resources they could tap into once their participation ended.	9-11 links sent over 14 days, at midday	"Hi Jon, the Stroke Foundation in Australia have put together a great blog post on how to improve your Problem Solving skills.<br><br>Click here to check it out when you get a chance!" (link)

Outcomes

Feasibility of trial design and procedures

Outcomes included the measurement of recruitment rates, including number of patients screened, eligible, consented, and excluded after screening; participant adherence to the intervention and usage (percentage of days using OnTrack, minutes of activity as recorded using OnTrack,

For peer review only - http://bmjopen.bmj.com/site/about/guidelines.xhtml

engagement with features in the OnTrack app); completion rates; and acceptability and reasons for decline/withdrawal.

NHS therapists responsible for screening and recruitment were invited to complete a survey to gather their feedback regarding acceptability of study procedures.

Clinical assessments

Clinical outcomes were collected to identify an appropriate primary outcome, and to estimate parameters for a sample size calculation for an RCT, these were collected at the start, halfway and end of participation (Table 2).

Table 2 - Outcome measure schedule

Concept	Assessment	Week performed
Patient Activation / Engagement	Patient Activation Measure (PAM)(24,25)	1, 8*, 14
Arm impairment	Fugl-Meyer Assessment for upper extremity (FMA-UE)**(26,27)	1, 8*, 14
Arm function	Upper-Extremity Motor Activity Log-14 (MAL)(28)	1, 8*, 14
Gross level of disability	modified Rankin Scale (mRS)(29)	1, 8*, 14
Arm pain	Visual Analogue Scale (VAS)(30)	1, 8*, 14
Cognitive impairment	Montreal Cognitive Assessment (MoCA)†(31,32)	1, 8*, 14
Arm neglect	Albert's Test (AT)(33)	1, 8*, 14
Quality of life	EQ-5D-5L(34)	1, 8*, 14
Activity baseline	Axivity AX3 usage on both arms	1, 14
System usability	System Usability Scale (SUS)(35)	14

* Performed at week 7 during Phase 2 of the study
** Not performed during Phase 2 due to inability to administer remotely
† A modified version was performed during Phase 2

Additional assessments

Activity baselines were gathered at weeks 1 and 14 of participation, study participants wore activity trackers (Axivity AX3) on both arms during waking hours (typically 12 hours/day) for 3-7 days to gather activity baseline data and allowing for left-right arm usage comparison.

The System Usability Scale (SUS)(35) was used to subjectively assess the usability of the OnTrack intervention.

Process evaluation

An independent process evaluation was conducted in parallel to this trial. The evaluation aimed to determine whether the intervention was delivered as intended and to understand the mechanisms of impact. Interviews were conducted with participants finishing the intervention period. These focused on their experience of using OnTrack and the different components of the intervention, as well as on their perceptions of the impact OnTrack had on their rehabilitation and ability to self-manage.

For peer review only - http://bmjopen.bmj.com/site/about/guidelines.xhtml

Sample size

The sample size was determined using guidelines that advocate a sample size between 12-30 for feasibility studies.(36) We aimed to recruit a minimum of 24 participants over the duration of the study which was in line with referral rates at the recruiting site.

Analysis

Analysis was performed on the study parameters and its implementation. We evaluated the usage of OnTrack as well as outcome measures and recorded changes over time.

Patterns of activity were analysed by day and hour of day. Comparisons between activity data and usage of different application features were created to understand their influence in activity output. Similarly, usage of individual features were recorded (e.g. engagement with messages, number of times activity stats were accessed, etc.). Furthermore, a comparison between activity minutes and performance in the different outcome measures was performed.

Data collected for the process evaluation was a combination of qualitative data from semi-structured interviews and observations of coaching sessions. At the study half-point, a workshop was conducted with the PPI group who analysed interview transcripts and made recommendations to refine the protocol.

Patient and public involvement (PPI)

A PPI group comprising three stroke survivors helped refine the intervention to the version used for this study. The group met a total of 4 times over the duration of the study, their time and travel were reimbursed according to INVOLVE (37) guidelines. The group supervised the development of all patient-facing material to ensure its clarity and accessibility. Members were trained by experienced researchers to participate in qualitative data analysis at the study's halfway point. They helped to refine themes and key messages arising from qualitative interviews. The PPI involvement plan was shared with Imperial College London's PPI 'Research Partners Group' to ensure the needs of the steering group were accounted for.

RESULTS

Recruitment

Recruitment took place between August 2019 and December 2020 with an imposed study suspension between March 2020 and August 2020 due to the public health emergency caused by the Covid-19 pandemic.

In total, 181 individuals were screened for eligibility with 37 meeting the inclusion criteria invited to participate (Figure 1). 24 participants were recruited with a response rate of 65%, of these, 15 (63%) were recruited before the study suspension and 9 (37%) between September and December 2020.

Participant characteristics

Data collected from participants during the initial assessment was available for 21 participants (11 females; mean age 61.1, range 33.5 - 82.5), three participants were lost to follow up before a session could be arranged. For the majority of participants (n=16, 76%) this was their first stroke, only one participant had had more than two previous strokes. Onset of the last stroke varied between 11 and

For peer review only - http://bmjopen.bmj.com/site/about/guidelines.xhtml

141 days with a mean of 42.2 days. 7 participants (33%) were impaired on their dominant arm. All participants owned or had experience with mobile phones but only 15 (71%) were smartphone users; 18 (86%) had WiFi at home. Although this was not a randomised study, participants' acceptance of randomisation was queried; 13 (62%) expressed they would have consented to the study regardless of whether they'd be allocated to a control or intervention group. Participant baseline characteristics can be seen on Table 3.

Table 3 - Participant characteristics at baseline (n=21)

Gender	11 female (52%)
Age (years); mean (SD); median (min, max)	61.1 (12.5); 60.5 (33.5 min, 82.5 max)
Ethnicity; n (%)	
White British	7 (33%)
White other	3 (14%)
Asian	5 (25%)
Black	3 (14%)
Other	2 (10%)
Prefer not to say	1 (5%)
Impaired arm	7 Right (33%)
Dominant arm	19 Right (90%)
Dominant arm impaired	7 Yes (33%)
Stroke onset (days); mean (SD); median (min, max)	42.2 (33.04); 33 (11 min, 141 max)
Type of stroke; n (%)	
Ischaemic	11 (52%)
Hemorrhagic	2 (10%)
Unknown	8 (38%)
First stroke	16 Yes (76%)
Comorbidities	15 Yes (71%)
Smoker	5 Yes (24%)
Smartphone user	15 Yes (71%)
WiFi at home	18 Yes (86%)
Amenable to randomisation	13 Yes (62%), 2 No (10%), 6 Don't know (29%)

SD = standard deviation

For peer review only

For peer review only - http://bmjopen.bmj.com/site/about/guidelines.xhtml

Adherence and retention

Of the 24 participants recruited, 21 (87%) took part in an initial session with researchers to gather baseline characteristics; 18 (75%) completed outcome measures and 17 (71%) went through to complete the activity baseline week. Of the 7 participants dropping out at this stage, researchers were unable to make contact with 5 of them; 1 was readmitted to hospital; and 1 decided to stop participation after feeling numbness on the arm.

17 (71%) participants started using OnTrack; 14 (58%) repeated outcome measures at halfway (week 7-8 of participation); and 12 (50%) completed the intervention period, repeated outcome measures, and completed the final activity baseline week. Of the 5 participants lost between starting OnTrack and finishing the study, 3 withdrew consent and 2 were lost to follow up. Reasons for withdrawing consent included one person who felt they had gained all they could from OnTrack and wanted to continue on their own; another felt that the intervention would not help them as they had too much on their mind; and another felt too anxious, fatigued and had a skin condition that was worsening.

From the 12 participants completing the intervention, one was unable to attend the final interview as they finished participation the week lockdown started in the UK and were living in sheltered accommodation making it impossible for the process evaluation team to make contact.

Adverse events

No serious adverse events were reported for this study. One participant was admitted to hospital at week 8 of participation for reasons unrelated to the study. Their participation was suspended for 4 weeks and later resumed in agreement between the participant, their family and the researchers.

On two occasions, participants reported feeling unwell and took one or two days off the intervention. They did not record their activity and no messages were sent to them during this period.

Two instances of data loss occurred, 5 weeks of data were lost for one participant and one week for another. Failure to sync data between the local devices (watch and smartphone) and the server accounted for these instances. This did not have an effect on the experience for the participants as they were able to access their data locally on their devices.

Acceptability and usability of OnTrack

Activity tracking

Compliance rate with activity tracking was measured by dividing the total number of days on the intervention by the total number of days the participant recorded their arm activity on the smartwatch. On average, participants were on the intervention for 83 days (min 76, max 84) and recorded their activity on 71 days (min 47, max 84), or the equivalent of 6.1 days per week for a compliance rate of 86% (Figure 2).

Furthermore an indication of usability and engagement with the intervention could be gathered by measuring the number of daily data interactions participants had using the applications on the smartphone and watch. Figure 3 shows how participants kept track of their activity data on the watch by looking at their activity data a mean of 7.5 times per day (min 4.5, max 12.3) and 3.5 times daily on the smartphone application (min 1, max 9.4).

A 63% mean increase in activity (min -1%, max 864%) could be observed in all participants when comparing the start and end of participation - or the equivalent of 4.3 more hours of activity per week. Participants partially attributed their motivation to do more activity to having a daily target of minutes

For peer review only - http://bmjopen.bmj.com/site/about/guidelines.xhtml

For peer review only

to achieve. On average, participants increased their target by 5% every session and managed an overall increase of 75% (min 18%, max 200%) between weeks 1 and 12, reaching their target on 3.1 days every week (Figure 4).

Messages and educational content

We measured the way participants engaged with messages, their views were explored during interviews. One participant was deemed cognitively unable to engage with messaging content, this participant was excluded from the messaging component of the intervention.

Overall, an average of 1.5 messages were sent per day and they were opened 1.3 times per day (Figure 5). Engagement with messages was lower than expected. One possible explanation is that messages were only sent to the smartphone and no notifications were received on the watch, if the participant didn't have the phone with them they would miss the message notification. In addition most participants felt that carrying the phone was sometimes a burden and so the OnTrack phone was left behind for long portions of the day.

Correlation between measured activity and application analytics

Correlations between recorded patient activity and application usage analytics were calculated in order to determine if these variables could be of interest for future analysis. There was a significant correlation between patients' activity and their daily activity target ($p < 0.001$, $R = 0.164$), however this correlation did not appear to be significant when compared to the weekly activity target ($p = 0.323$, $R=-0.035$). There was no significant association with the number of times patients saw their activity graph on their watch ($R = -0.035$, $p=0.323$). We did not identify a significant difference (Wilcoxon Signed Ranks Test (38), $p=0.28$) in activity minutes based on whether or not patients accepted their intention for the day (Yes = 91.7 minutes, No answer = 64.4 minutes). Finally, there were positive and significant correlations with the number of times patients viewed messages sent ($p < 0.001$, $R = 0.201$), and the number of links opened ($p = 0.002$, $R = 0.107$).

Views on the intervention and study procedures

Participant views

Participants provided feedback on the study procedures, they felt that recruitment procedures were acceptable and that the information they received was appropriate. Some participants felt that the amount of assessments conducted could be tiring and recommended either reducing them or conducting them over two or more sessions. The activity sensors worn on both wrists during the first and last weeks of participation were uncomfortable for some participants and some required help to put them on.

An in-depth analysis on the experience of participating and using the intervention is reported in a process evaluation publication. In summary, participants felt OnTrack was working as expected and intended. They expressed feeling well supported to use the technology, even without prior experience. They reported feeling that the activity targets were motivating, although they would welcome more specific activity recommendations alongside targets. All participants mentioned that the quality of the coaching was an important part of creating a positive experience. Participants found remote sessions acceptable and reported valuing the contact during lockdown.

For peer review only - http://bmjopen.bmj.com/site/about/guidelines.xhtml

Therapist views

Therapists contributing to screening and recruitment completed an online survey exploring three themes: the time spent performing study procedures; the intervention and the potential benefits to stroke survivors; and views on patient engagement needed to use OnTrack. 6 of 13 therapists completed the survey (46.2% completion rate).

Overall, therapists felt that the time they spent in study procedures was in line with expectations, and that the tasks performed were acceptable.

On The potential benefits of the intervention, therapists thought that the intervention could be beneficial in general terms and that it could be of most benefit to stroke survivors with motor and sensory impairment. Benefits to patients with arm neglect were less clear but tended to the positive.

Therapists agreed that the intervention could help motivate patients into performing more activities with their impaired arm and support them in the self-management of their recovery. However, some respondents felt that patients who are less engaged with their recovery may not fully benefit from the intervention.

Clinical outcomes

A wide range of outcome measures were collected (Table 4) in order to assess their suitability and feasibility for use during a future definitive study. Pandemic restrictions brought forward new considerations; for example, despite being a widely used assessment for arm impairment after stroke, the FMA-UE was only used during Phase 1 of the study as it was impossible to perform once the study moved to a remote format of delivery. On the other hand, the MAL can be performed remotely and has proven to be a reliable and valid measure of outcome from rehabilitation and of functional status in patients with arm impairment post stroke.(28) An estimate sample size of 46 was calculated using the MAL's reported minimal clinically important difference (39) with 90% power and two-sided alpha of 5%.

A member of the research team was responsible for completing all assessments either in person (Phase 1) or remotely (Phase 2).

Table 4 - Outcome measures at baseline and follow-up points, mean and (SD)

Outcome	Baseline	Halfway*	14 weeks
PAM	69.7 (17.8)	65.8 (14.8)	68.1 (10.3)
FMA-UE**	37.7 (17.2)	39.0 (20.1)	36.4 (22.4)
MAL***	2.00 (1.4) | 2.26 (1.5)	2.94 (1.2) | 3.09 (1.2)	3.24 (1.3) | 3.17 (1.2)
mRS	2.8 (1.1)	2.5 (0.8)	1.9 (0.9)
VAS (pain)	0.8 (1.0)	2.5 (1.9)	2.8 (2.3)
MoCA†	22.6 (6.9)	24.0 (5.1)	25.3 (1.0)
MoCA††	17.8 (3.4)	19.0 (1.9)	18.3 (2.4)
AT	0.2 (0.4)	0.1 (0.3)	0.1 (0.3)
EQ-5D-5L index	0.462 (0.3)	0.585 (0.1)	0.606 (0.2)

For peer review only - http://bmjopen.bmj.com/site/about/guidelines.xhtml

EQ-5D-5L VAS	57.3 (20.7)	72.7 (9.8)	74.2 (15.1)
SUS			84.6 (13.1)

*8 weeks for Phase 1 participants; 7 weeks for Phase 2.
** Only performed during Phase 1. Participants who started in Phase 1 but finished their participation after lock down, did not complete this measure subsequently.
*** How much score | How well score
† Full version applied before lockdown measures (scores out of 30)
†† Telephone version applied after lockdown measures (scores out of 22)
PAM - Patient Activation Measure; FMA-UE - FuglMeyer Upper Extremity; MAL - Motor Activity Log; mRS - Modified Rankin Scale; VAS - Visual Analogue Scale (pain); MoCA - Montreal Cognitive Assessment; AT - Albert's Test; EQ-5D-5L - EuroQol; SUS - System Usability Scale.

Correlations between measured activity and outcome measures

Several outcome variables were modelled (Table 5). Individual models were created for each of the following variables: left-right arm usage ratio improvement, which measures whether or not the patients' difference in arm usage improved by the end of the study (this model also included an additional independent variable which captured whether or not the patients' impaired arm was their dominant arm, model fit $p < 0.001$), PAM (model fit $p = 0.002$), VAS (model fit $p < 0.001$), MAL - How much score (model fit $p < 0.001$), MAL - how well score (model fit $p < 0.001$), mRS (model fit $p < 0.001$), MoCA (model fit $p = 0.158$), EQ-5D-5L index (model fit $p < 0.001$), EQ-5D-5L VAS (model fit $p < 0.001$).

Table 5 - Outcome measures model estimates

Models were created by using the outcome variable as a dependent variable, and using the variables listed under the 'Model variables' as covariates. 'Activity' quantifies segments of 30-minutes of daily activity, 'Time' determines the number of days since each patient started recording their activity, and 'Dominant arm' was used to determine whether or not the patient suffered a stroke on their dominant arm.

Outcome	Model adjusted R^2	Model fit p value	Model variables	Estimates	Estimate p value
Left-Right ratio improvement* (Ref. category: No improvement)	0.169†	< 0.001	Activity<br>Time<br>Dominant arm (Ref. category: No)	1.005††<br>0.995††<br>6.684††	< 0.001<br>0.116<br>< 0.001
PAM	0.012	0.002	Activity<br>Time	-0.653<br>-0.022	0.002<br>0.290
VAS	0.135	< 0.001	Activity<br>Time	-0.056<br>0.29	0.025<br>0.000
MAL - how much	0.452	< 0.001	Activity<br>Time	0.304<br>0.012	< 0.001<br>< 0.001
MAL- how well	0.433	< 0.001	Activity<br>Time	0.324<br>0.001	< 0.001<br>< 0.001
mRS	0.267	< 0.001	Activity<br>Time	-0.161<br>-0.007	< 0.001<br>< 0.001
MoCA	0.002	0.158	Activity<br>Time	0.002<br>0.000	0.272<br>0.160
EQ-5D-5L index	< 0.001	0.161	Activity<br>Time	0.020<br>0.002	< 0.001<br>< 0.001
EQ-5D-5L VAS	0.226	<0.001	Activity<br>Time	1.252<br>0.241	< 0.001<br>< 0.001

For peer review only - http://bmjopen.bmj.com/site/about/guidelines.xhtml

* Left-right ratio improvement was measured using a logistic regression model, as opposed to other variables which were modeled using a linear model.
† Pseudo R^2 (McFadden)
†† Estimated for the left-right ratio improvement are show as add ratios

Fidelity of intervention delivery

Fidelity of delivery was assessed through the observation of coaching sessions. Observations during the first half of the study identified that the delivery of the coaching sessions was inconsistent with how participants were experiencing their recovery. This resulted in the intervention team consistently deviating from the coaching plan in response to participant needs.

A workshop involving the PPI group was planned at the study half-way point to refine the delivery of the intervention for Phase 2. A summary of findings from the half-way workshop suggested that overall, the OnTrack intervention was working as intended, however the following points were observed:

- Participants reported a lack of clarity about whether the focus of the intervention was to improve hand and arm movement, or to improve activity more generally.
- In coaching sessions, the discussions relating to the activity target were distinct from those relating to individual functional goals, and the self-management strategies were not easy to observe.
- The activity targets were regarded as motivating.
- Participants wanted more specific activities as targets, e.g. holding a cup, making toast.
- Participants valued facts and useful information in messages.
- The coaches were seen as central – the language and strategies they use is key to promoting self-management.

In response to these findings, a more fluid and flexible approach to coaching was developed. The new guide included prompts for supporting self-management during all sessions, as opposed to being session specific, it encouraged focusing more on discussing the meaningful activities that participants would like to use their arms for, and using the activity data to support this (a table describing these changes is provided as a supplementary file).

Delivery of the intervention during Phase 2 was affected by the coronavirus pandemic. All sessions were delivered remotely, participants stated that this was acceptable and welcomed the contact with coaches during lockdown.

DISCUSSION

Summary of principal findings

This study aimed to assess the acceptability of a novel digital intervention to support arm and hand rehabilitation after stroke and the feasibility of conducting a definitive trial into its effectiveness. The study met its objectives in assessing the acceptability and feasibility of the intervention and trial procedures. Despite being affected by the pandemic, recruitment to the trial was in line with pre-study expectations, as well as participant retention rates. Appropriate outcome measures and sample size estimations for an RCT were analysed. Qualitative data were gathered showing that the intervention was usable and acceptable to study participants, but most importantly, it highlighted the components of OnTrack that were perceived as most valuable to them.

For peer review only - http://bmjopen.bmj.com/site/about/guidelines.xhtml

Strengths and weaknesses of the study

Findings from this study have shown a positive way in which wearable technology and self-management coaching could be combined to improve rehabilitation outcomes by helping participants identify opportunities for self-practice and become more engaged in their recovery.

The coronavirus pandemic significantly affected the running of this study. In the first instance, a suspension in recruitment was necessary, followed by an amendment to the recruitment and consenting procedures. In addition, the adaptation to lockdown restrictions accelerated the need for remote delivery of OnTrack, this however had little impact on participants' experiences.

The independent process evaluation provided an opportunity to understand the different mechanisms of impact. Allowing for refinement at the half-way point enabled the researchers to incorporate feedback to modify the way OnTrack was delivered. The engagement of the PPI group enabled this refinement to be implemented through a user-centred process. However, the process evaluation also found that the messaging component of the intervention received mixed reviews by participants. On the one hand, the practical information sent was perceived as valuable, but the tone of voice and frequency of other messages was questioned by some participants. This should be revised for a future version of OnTrack.

Due to pragmatic reasons, the study could not cater for longer term participant follow-up. During interviews, participants expressed they would value a check some time after the program ended. A future study should consider incorporating longer term follow-up to understand if participants continue to apply self-management principles.

Unanswered questions and future research

Whilst randomisation was not possible during this study, participants were asked if they would be willing to participate in a randomised study. Most participants said that randomisation would not influence their decision to participate, however more consideration is needed to understand the appropriate design of control and intervention arms.

The messaging component of this intervention needs further refinement and co-design in collaboration with stroke survivors. Whilst, for the most part, the messages are seen as a valuable component, their design requires a better understanding of the content, context, frequency and tone of voice in which they are delivered.

The intervention was delivered over 12 weeks, however questions remain as to whether OnTrack could be delivered over shorter or longer periods and how this would affect the design of the coaching component. The coaching component itself requires further thinking in how it may be delivered at scale, the training required, and other tools needed to support this role. For this trial, coaching guidelines were used and coaches had access to a digital dashboard showing participants' activity. Future development and studies should focus on understanding these components from the perspective of therapy teams.

ORIGINAL PROTOCOL

https://bmjopen.bmj.com/content/10/3/e034936

For peer review only - http://bmjopen.bmj.com/site/about/guidelines.xhtml

AUTHOR CONTRIBUTIONS

AD was Principal Investigator and grant holder and oversaw the project along with DD. GF, EG and LH developed the intervention and conceived of the study. GF, EG, and FJ initiated the study design and ET and ML helped with further refinement. ALJ led on PPI. EG and GF were responsible for delivering the intervention and data collection. JC had participant screening oversight. FJ and ET were responsible for the process evaluation. ML and RFC conducted the primary statistical analysis. All authors contributed to this manuscript and approved the final version.

FUNDING STATEMENT

This study was funded by the NIHR Imperial Biomedical Research Centre (BRC), grant 1215-20013. The views expressed are those of the author(s) and not necessarily those of the NIHR or the Department of Health and Social Care.

DATA STATEMENT

Requests for analyses of anonymised data from this trial should be directed to the corresponding author.

CONFLICT OF INTERESTS

None.

ACKNOWLEDGMENTS

The authors would like to acknowledge the contributions of all the members of the PPI group; all participants involved; Peter Wilding, Meenakshi Nayar, and Davina Richardson and therapy teams at ICHT; therapy teams at CLCH; Ruth Nicholson for her support in ethics applications and procedures; Steve McAteer and Eleni Daniels for their guidance on the BRC program; Dr Hendramoorthy Maheswaran for his help reviewing the manuscript.

ETHICS STATEMENT

Approval granted by the NHS Health Research Authority, Health and Care Research Wales, and the London—Surrey Research Ethics Committee (ref. 19/LO/0881).

REFERENCES

(1) WHO. *The Atlas of Heart Disease and Stroke.* Available from: https://www.who.int/cardiovascular_diseases/resources/atlas/en/ [Accessed 04 Oct 2019].

(2) Stroke Association. *State of the Nation: stroke statistics.* Stroke Association. 2018.

(3) Patel, A., Berdunov, V., King, D., Quayyum, Z., Wittenberg, R. & Knapp, M. *Current, future and avoidable costs of stroke in the UK* . Stroke Association. 2017.

(4) *Shaping stroke research to rebuild lives: The Stroke Priority Setting Partnership results for investment.* 2021.

For peer review only - http://bmjopen.bmj.com/site/about/guidelines.xhtml

(5) Care Quality Commission. *Supporting life after stroke: a review of services for people who have had a stroke and their carers.* London: Care Quality Commission. 2011.

(6) Bernhardt J, Dewey H, Thrift A, Donnan G. Inactive and Alone: Physical Activity Within the First 14 Days of Acute Stroke Unit Care. *Stroke.* 2004; 35 (4): 1005-1009. Available from: doi: 10.1161/01.STR.0000120727.40792.40 Available from: http://stroke.ahajournals.org/cgi/content/abstract/35/4/1005 .

(7) Kunkel D, Fitton C, Burnett M, Ashburn A. Physical inactivity post-stroke: a 3-year longitudinal study. *Disability & Rehabilitation.* 2015; 37 (4): 304-310. Available from: doi: 10.3109/09638288.2014.918190 Available from: http://www.tandfonline.com/doi/abs/10.3109/09638288.2014.918190 .

(8) Pollock A, Farmer SE, Brady MC, Langhorne P, Mead GE, Mehrholz J, et al. Interventions for improving upper limb function after stroke. *The Cochrane database of systematic reviews.* 2014; 2014 (11): CD010820. Available from: doi: 10.1002/14651858.CD010820.pub2 Available from: https://www.ncbi.nlm.nih.gov/pubmed/25387001 .

(9) Royal College of Physicians. *Clinical guide for the management of stroke patients during the coronavirus pandemic.* 2020.

(10) *Coronavirus and stroke (Stroke Association).* Available from: https://www.stroke.org.uk/finding-support/coronavirus-covid-19-and-stroke.

(11) Salter K, Hellings C, Foley N, Teasell R. The Experience of Living with Stroke: A Qualitative Meta-Synthesis. *Journal of Rehabilitation Medicine.* 2008; 40 (8): 595-602. Available from: doi: 10.2340/16501977-0238 Available from: http://www.ingentaconnect.com/content/mjl/sreh/2008/00000040/00000008/art00003 .

(12) Buckland D. Leaving hospital is 'like falling off a cliff' for stroke survivors. *Raconteur.* APRIL 27, 2017 . Available from: https://www.raconteur.net/healthcare/leaving-hospital-is-like-falling-off-a-cliff-for-stroke-survivors [Accessed Oct 04 2019].

(13) McKevitt C, Fudge N, Redfern J, Sheldenkar A, Crichton S, Rudd AR, et al. Self-Reported Long-Term Needs After Stroke. *Stroke.* 2011; 42 (5): 1398-1403. Available from: doi: 10.1161/STROKEAHA.110.598839 Available from: https://www.ncbi.nlm.nih.gov/pubmed/21441153

(14) Woodman P, Riazi A, Pereira C, Jones F. Social participation post stroke: a meta-ethnographic review of the experiences and views of community-dwelling stroke survivors. *Disability & Rehabilitation.* 2014; 36 (24): 2031-2043. Available from: doi: 10.3109/09638288.2014.887796 Available from: http://www.tandfonline.com/doi/abs/10.3109/09638288.2014.887796 .

(15) Jones F, Gombert K, Honey S, Cloud G, Harris R, Macdonald A, et al. Addressing inactivity after stroke: The Collaborative Rehabilitation in Acute Stroke (CREATE) study. *International journal of stroke.* 2021; 16 (6): 669-682. Available from: doi: 10.1177/1747493020969367 Available from: https://journals.sagepub.com/doi/full/10.1177/1747493020969367 .

(16) Esmonde T, McGinley J, Wittwer J, Goldie P, Martin C. Stroke rehabilitation: patient activity during non-therapy time. *Australian Journal of Physiotherapy.* 1997; 43 (1): 43-51. Available from: doi: 10.1016/S0004-9514(14)60401-3 Available from: https://www.sciencedirect.com/science/article/pii/S0004951414604013 .

(17) Jones F, Gage H, Drummond A, Bhalla A, Grant R, Lennon S, et al. Feasibility study of an integrated stroke self-management programme: a cluster-randomised controlled trial. *BMJ Open.* 2016; 6 (1): e008900. Available from: doi: 10.1136/bmjopen-2015-008900 Available from: http://dx.doi.org/10.1136/bmjopen-2015-008900 .

(18) Harwood M, Weatherall M, Talemaitoga A, Barber PA, Gommans J, Taylor W, et al. Taking charge after stroke: promoting self-directed rehabilitation to improve quality of life – a randomized controlled trial. *Clinical Rehabilitation.* 2012; 26 (6): 493-501. Available from: doi: 10.1177/0269215511426017 Available from: https://journals.sagepub.com/doi/full/10.1177/0269215511426017 .

For peer review only - http://bmjopen.bmj.com/site/about/guidelines.xhtml

(19) Kendall E, Catalano T, Kuipers P, Posner N, Buys N, Charker J. Recovery following stroke: The role of self-management education. *Social Science & Medicine.* 2007; 64 (3): 735-746. Available from: doi: 10.1016/j.socscimed.2006.09.012 Available from: https://www.sciencedirect.com/science/article/pii/S0277953606004801 .

(20) Grady PA, Gough LL. Self-Management: A Comprehensive Approach to Management of Chronic Conditions. *American journal of public health.* 2014; 104 (8): e25-e31. Available from: doi: 10.2105/AJPH.2014.302041 Available from: https://www.ncbi.nlm.nih.gov/pubmed/24922170 .

(21) Fusari G, Gibbs E, Hoskin L, et al. Protocol for a feasibility study of OnTrack: a digital system for upper limb rehabilitation after stroke. *BMJ Open* 2020;10:e034936. doi:10.1136/ bmjopen-2019-034936

(22) Bridges Self-Management Limited. *Bridges Self-Management.* Available from: http://www.bridgesselfmanagement.org.uk/ [Accessed 07 Oct 2019].

(23) Medical Research Institute of New Zealand. *Taking Charge After Stroke (TaCAS).* Available from: https://mrinz.ac.nz/programmes/stroke/ [Accessed 07 oct 2019].

(24) Hibbard JH, Stockard J, Mahoney ER, Tusler M. Development of the Patient Activation Measure (PAM): Conceptualizing and Measuring Activation in Patients and Consumers. *Health Services Research.* 2004; 39 (4p1): 1005-1026. Available from: doi: 10.1111/j.1475-6773.2004.00269.x Available from: https://onlinelibrary.wiley.com/doi/abs/10.1111/j.1475-6773.2004.00269.x .

(25) Roberts NJ, Kidd L, Dougall N, Patel IS, McNarry S, Nixon C. Measuring patient activation: The utility of the Patient Activation Measure within a UK context—Results from four exemplar studies and potential future applications. *Patient Education and Counseling.* 2016; 99 (10): 1739-1746. Available from: doi: 10.1016/j.pec.2016.05.006 Available from: https://www.clinicalkey.es/playcontent/1-s2.0-S0738399116301938 .

(26) Sahlgrenska Academy Institute of Neuroscience and Physiology, University of Gothenburg. *Fugl-Meyer.* Available from: https://neurophys.gu.se/english/departments/clinical_neuroscience_and_rehabilitation/rehabilitation-medicine/fugl-meyer [Accessed 04 Oct 2019].

(27) Pamela W Duncan, Martha Propst, Steven G Nelson. Reliability of the Fugl-Meyer Assessment of Sensorimotor Recovery Following Cerebrovascular Accident. *Physical Therapy.* 1983; 63 (10): 1606-1610. Available from: doi: 10.1093/ptj/63.10.1606 Available from: http://ptjournal.apta.org/content/63/10/1606.abstract .

(28) Uswatte G, Taub E, Morris D, Vignolo M, McCulloch K. Reliability and Validity of the Upper-Extremity Motor Activity Log-14 for Measuring Real-World Arm Use. *Stroke.* 2005; 36 (11): 2493-2496. Available from: doi: 10.1161/01.STR.0000185928.90848.2e Available from: http://stroke.ahajournals.org/cgi/content/abstract/36/11/2493 .

(29) Quinn TJ, Dawson J, Walters MR, Lees KR. Exploring the Reliability of the Modified Rankin Scale. *Stroke.* 2009; 40 (3): 762-766. Available from: doi: 10.1161/STROKEAHA.108.522516 Available from: http://stroke.ahajournals.org/cgi/content/abstract/40/3/762 .

(30) Jensen MP, Karoly P, Braver S. The measurement of clinical pain intensity: a comparison of six methods. *Pain.* 1986; 27 (1): 117-126. Available from: doi: 10.1016/0304-3959(86)90228-9 Available from: https://www.sciencedirect.com/science/article/pii/0304395986902289 .

(31) Nasreddine ZS, Phillips NA, Bédirian V, Charbonneau S, Whitehead V, Collin I, et al. The Montreal Cognitive Assessment, MoCA: A Brief Screening Tool For Mild Cognitive Impairment. *Journal of the American Geriatrics Society.* 2005; 53 (4): 695-699. Available from: doi: 10.1111/j.1532-5415.2005.53221.x Available from: https://onlinelibrary.wiley.com/doi/abs/10.1111/j.1532-5415.2005.53221.x .

(32) *MoCA Test - Remote MoCA Testing.* Available from: https://www.mocatest.org/remote-moca-testing/ [Accessed 09 September 2021].

For peer review only - http://bmjopen.bmj.com/site/about/guidelines.xhtml

(33) Fullerton KJ, McSherry D, Stout RW. Albert's test: a neglected test of perceptual neglect. *Lancet (London, England).* 1986; 1 (8478): 430. Available from: https://www.ncbi.nlm.nih.gov/pubmed/2868349 .

(34) EuroQol - a new facility for the measurement of health-related quality of life. *Health policy.* 1990; 16 (3): 199-208. Available from: doi: 10.1016/0168-8510(90)90421-9 Available from: https://www.sciencedirect.com/science/article/pii/0168851090904219 .

(35) SUS: A 'Quick and Dirty' Usability Scale. *Usability Evaluation In Industry.* CRC Press; 1996. pp. 207-212.

(36) Billingham SAM, Whitehead AL, Julious SA. An audit of sample sizes for pilot and feasibility trials being undertaken in the United Kingdom registered in the United Kingdom Clinical Research Network database. *BMC medical research methodology.* 2013; 13 (1): 104. Available from: doi: 10.1186/1471-2288-13-104 Available from: https://www.ncbi.nlm.nih.gov/pubmed/23961782 .

(37) National Institute for Health Research. *About INVOLVE.* Available from: https://www.invo.org.uk/about-involve/ [Accessed 04 Oct 2019].

(38) Scheff SW. Nonparametric Statistics. *Fundamental Statistical Principles for the Neurobiologist.* Elsevier Inc; 2016. pp. 157-182.

(39) Lang CE, Edwards DF, Birkenmeier RL, Dromerick AW. Estimating Minimal Clinically Important Differences of Upper-Extremity Measures Early After Stroke. *Archives of physical medicine and rehabilitation.* 2008; 89 (9): 1693-1700. Available from: doi: 10.1016/j.apmr.2008.02.022 Available from: https://search.datacite.org/works/10.1016/j.apmr.2008.02.022 .

FIGURE LEGEND

Figure 1 - CONSORT flow diagram

Figure 2 - Average days recording per week (min, max)

Figure 3 - Average daily data views on phone and watch

Figure 4 - Weekly activity, target, and number of days the target was reached

Figure 5 - Overall messages sent / opened (daily average)

For peer review only

For peer review only - http://bmjopen.bmj.com/site/about/guidelines.xhtml

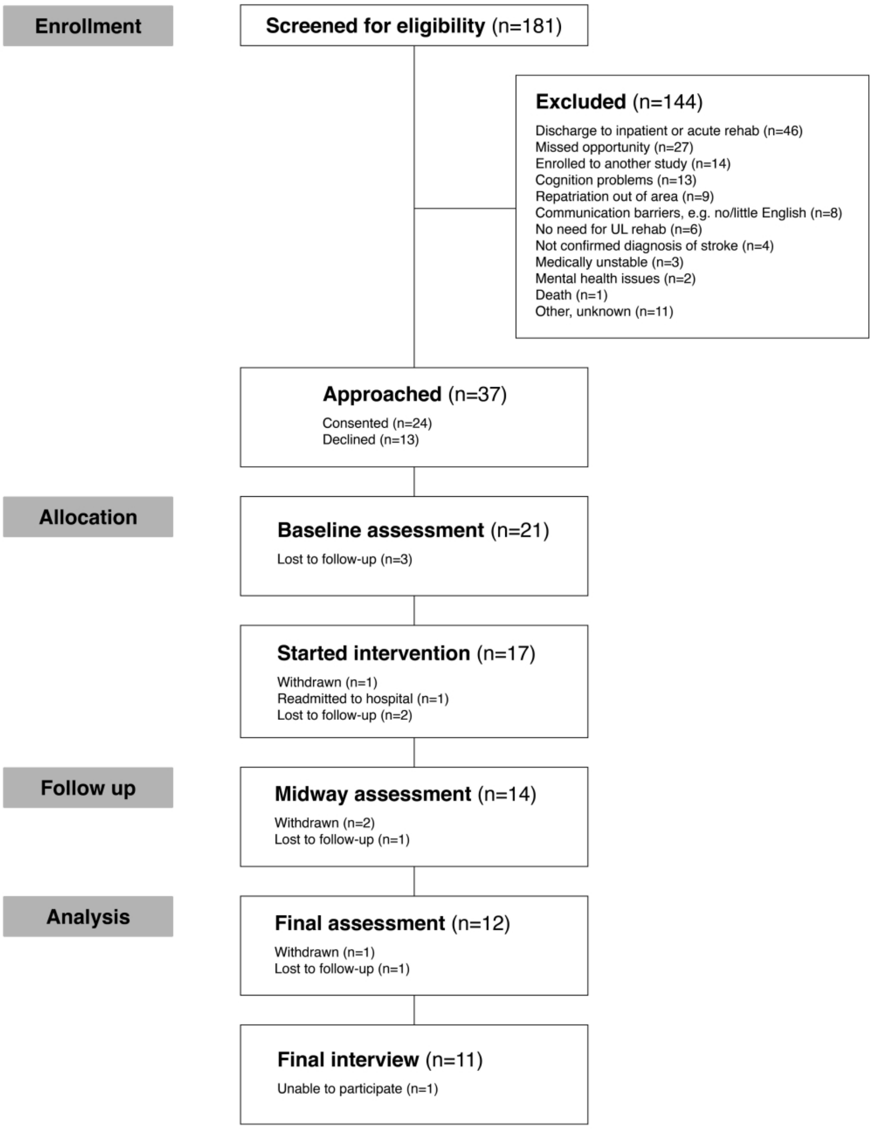

Figure 1 - CONSORT flow diagram

1609x2063mm (72 x 72 DPI)

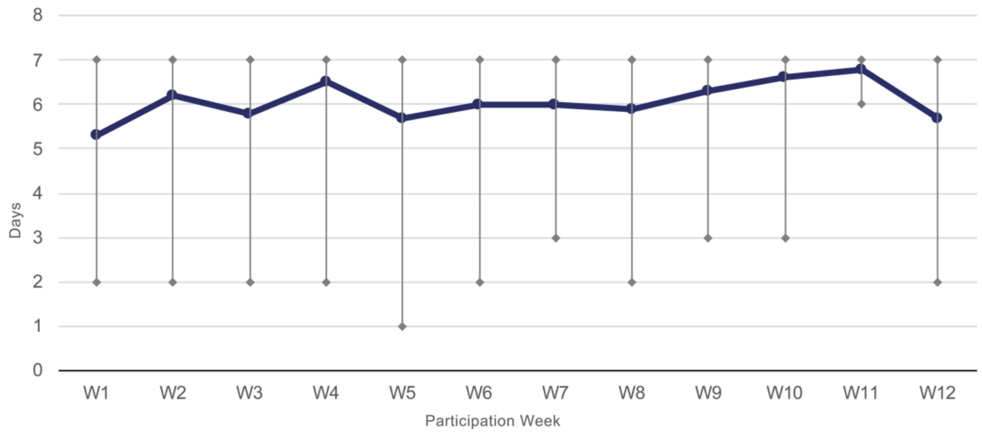

Figure 2 - Average days recording per week (min, max)

115x51mm (600 x 600 DPI)

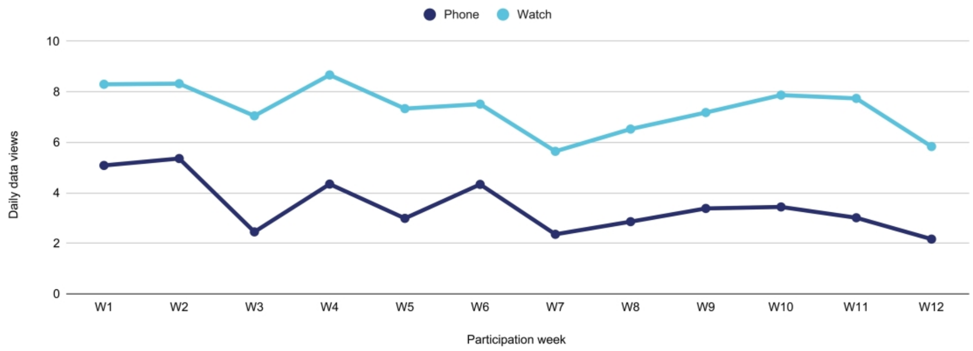

Figure 3 - Average daily data views on phone and watch

173x65mm (600 x 600 DPI)

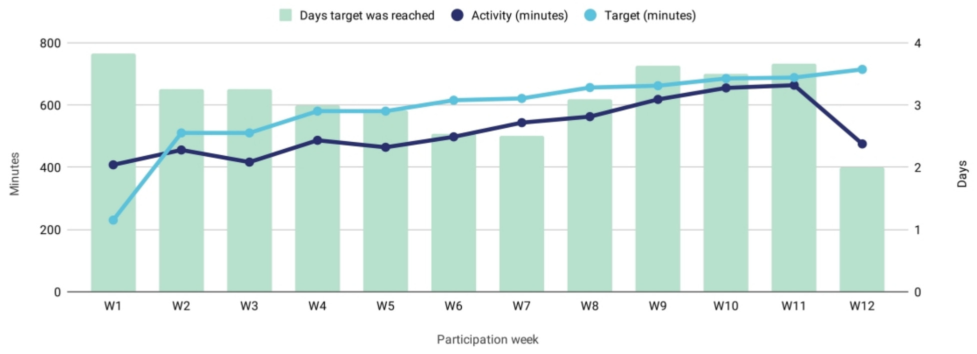

Figure 4 - Weekly activity, target, and number of days the target was reached

173x65mm (600 x 600 DPI)

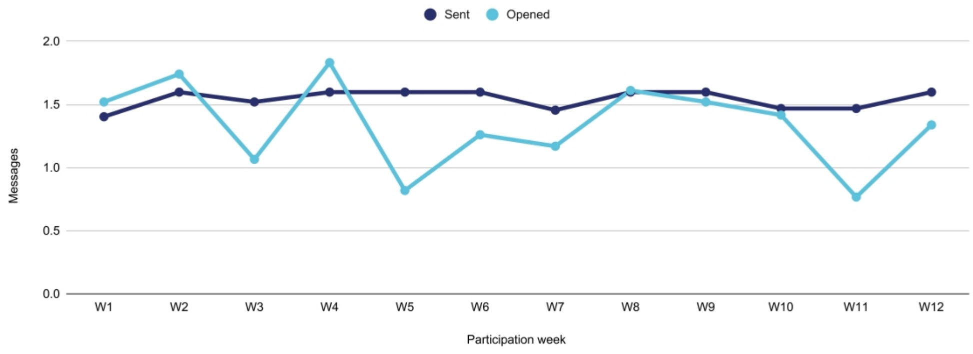

Figure 5 - Overall messages sent / opened (daily average)

173x65mm (600 x 600 DPI)

**Supplementary file 1 - Phase 1 and Phase 2 coaching structures**			
**Coaching delivery - Study phase 1**			
**Week**	**Session**	**Coaching phase**	**Coaching guidelines**
W1	Baseline assessment	*No coaching*	
W2	Coaching session 1	Onboarding	Introduction to OnTrack / purpose, value, practical issues
W3	Coaching session 2	Problem solving	Encouraging people to discuss areas where they struggle, then facilitate coming up with ideas and strategies around problems faced during daily life situations.
W4			
W5	Coaching session 3	Self-discovery	Finding out new ways of doing things, trying out different activities, so that any attempt to try and find a new way around something is seen as a positive.
W6			
W7	Coaching session 4	Goal setting	Working with participants to identify, plan and execute steps towards a goal regardless of size and ambition. The aim is to help participants learn and get comfortable with this process under their terms.<br><br>Outcome measure collection.
W8	Halfway assessment		
W9			
W10	Coaching session 5	Reflection	Looking back at achievements and failures to understand what worked and what didn't in order to adjust if necessary and look forward to where they want to be.
W11			
W12	Coaching session 6	Signposting	Using goals and aspirations to help participants create links that encourage social participation, community integration and/or peer support.
W13			
W14	Final assessment	*No coaching*	
**Coaching delivery - Study phase 2**			
**Week**	**Session**	**Coaching phase**	**Coaching guidelines**
W1	Baseline assessment	*No coaching*	Initial contact; conversation about participant's background and present situation.
W2	Coaching session 1	Introduction	Focus on creating a habit of using OnTrack and charging devices. Setting the scene for coaching.
W3	Coaching session 2	OnTrack coaching	In-depth conversations about participants' aspirations and what's important to them.<br><br>Discussion to assess participants' understanding of stroke and rehab mechanisms, identify topics of curiosity, signpost as necessary.<br><br>Use of self-management principles (Problem Solving, Self-
W4			
W5	Coaching session 3		

For peer review only

W6			discovery, Goal Setting, and Reflection) and rehab guidelines to focus conversations towards reaching an agreed activity plan (however small).<br><br>Make connections between activity performed and data recorded.
W7	Halfway assessment		
W8			
W9	Coaching session 4		
W10			
W11	Coaching session 5		
W12			
W13	Coaching session 6	Signposting	Signpost to relevant resources that can be accessed by participants going forward.
W14	Final assessment	*No coaching*	Closure; last query to ensure participants feel confident to continue their rehab on their own.

For peer review only

Reporting checklist for quality improvement in health care.

Based on the SQUIRE guidelines.

Instructions to authors

Complete this checklist by entering the page numbers from your manuscript where readers will find each of the items listed below.

Your article may not currently address all the items on the checklist. Please modify your text to include the missing information. If you are certain that an item does not apply, please write "n/a" and provide a short explanation.

Upload your completed checklist as an extra file when you submit to a journal.

In your methods section, say that you used the SQUIREreporting guidelines, and cite them as:

Ogrinc G, Davies L, Goodman D, Batalden P, Davidoff F, Stevens D. SQUIRE 2.0 (Standards for QUality Improvement Reporting Excellence): revised publication guidelines from a detailed consensus process

		Reporting Item	Page Number
**Title**			
	#1	Indicate that the manuscript concerns an initiative to improve healthcare (broadly defined to include the quality, safety, effectiveness, patientcenteredness, timeliness, cost, efficiency, and equity of healthcare)	1
**Abstract**			
	#02a	Provide adequate information to aid in searching and indexing	1
	#02b	Summarize all key information from various sections of the text using the abstract format of the intended publication or a structured summary such as: background, local problem, methods, interventions, results, conclusions	1
**Introduction**			
Problem description	#3	Nature and significance of the local problem	3

For peer review only

Available knowledge	#4	Summary of what is currently known about the problem, including relevant previous studies	3
Rationale	#5	Informal or formal frameworks, models, concepts, and / or theories used to explain the problem, any reasons or assumptions that were used to develop the intervention(s), and reasons why the intervention(s) was expected to work	3
Specific aims	#6	Purpose of the project and of this report	3
**Methods**			
Context	#7	Contextual elements considered important at the outset of introducing the intervention(s)	4
Intervention(s)	#08a	Description of the intervention(s) in sufficient detail that others could reproduce it	4
Intervention(s)	#08b	Specifics of the team involved in the work	4
Study of the Intervention(s)	#09a	Approach chosen for assessing the impact of the intervention(s)	4
Study of the Intervention(s)	#09b	Approach used to establish whether the observed outcomes were due to the intervention(s)	4
Measures	#10a	Measures chosen for studying processes and outcomes of the intervention(s), including rationale for choosing them, their operational definitions, and their validity and reliability	6
Measures	#10b	Description of the approach to the ongoing assessment of contextual elements that contributed to the success, failure, efficiency, and cost	6
Measures	#10c	Methods employed for assessing completeness and accuracy of data	6
Analysis	#11a	Qualitative and quantitative methods used to draw inferences from the data	7
Analysis	#11b	Methods for understanding variation within the data, including the effects of time as a variable	7
Ethical considerations	#12	Ethical aspects of implementing and studying the intervention(s) and how they were addressed, including, but	2

For peer review only

		not limited to, formal ethics review and potential conflict(s) of interest	
**Results**			
	#13a	Initial steps of the intervention(s) and their evolution over time (e.g., time-line diagram, flow chart, or table), including modifications made to the intervention during the project	7
	#13b	Details of the process measures and outcome	8
	#13c	Contextual elements that interacted with the intervention(s)	8
	#13d	Observed associations between outcomes, interventions, and relevant contextual elements	10
	#13e	Unintended consequences such as unexpected benefits, problems, failures, or costs associated with the intervention(s).	9
	#13f	Details about missing data	9
**Discussion**			
Summary	#14a	Key findings, including relevance to the rationale and specific aims	16
Summary	#14b	Particular strengths of the project	16
Interpretation	#15a	Nature of the association between the intervention(s) and the outcomes	16
Interpretation	#15b	Comparison of results with findings from other publications	16
Interpretation	#15c	Impact of the project on people and systems	16
Interpretation	#15d	Reasons for any differences between observed and anticipated outcomes, including the influence of context	16
Interpretation	#15e	Costs and strategic trade-offs, including opportunity costs	n/a clinical feasibility and acceptability study
Limitations	#16a	Limits to the generalizability of the work	16
Limitations	#16b	Factors that might have limited internal validity such as confounding, bias, or imprecision in the design, methods, measurement, or analysis	16

For peer review only

Limitations	#16c	Efforts made to minimize and adjust for limitations	n/a clinical feasibility and acceptability study
Conclusion	#17a	Usefulness of the work	16
Conclusion	#17b	Sustainability	n/a clinical feasibility and acceptability study
Conclusion	#17c	Potential for spread to other contexts	n/a clinical feasibility and acceptability study
Conclusion	#17d	Implications for practice and for further study in the field	16
Conclusion	#17e	Suggested next steps	16
**Other information**			
Funding	#18	Sources of funding that supported this work. Role, if any, of the funding organization in the design, implementation, interpretation, and reporting	17

The SQUIRE 2.0 checklist is distributed under the terms of the Creative Commons Attribution License CC BY-NC 4.0. This checklist was completed on 10. February 2022 using https://www.goodreports.org/, a tool made by the EQUATOR Network in collaboration with Penelope.ai

For peer review only

BMJ Open

What is the feasibility and patient acceptability of a digital system for arm and hand rehabilitation after stroke? A mixed methods, single-arm feasibility study of the ‘OnTrack’ intervention for hospital and home use.

Journal:	*BMJ Open*
Manuscript ID	bmjopen-2022-062042.R1
Article Type:	Original research
Date Submitted by the Author:	12-Aug-2022
Complete List of Authors:	Fusari, Gianpaolo; Imperial College London, Helix Centre<br>Gibbs, Ella; Imperial College London, Helix Centre<br>Hoskin, Lily; Imperial College London, Helix Centre<br>Lawrence-Jones, Anna; Imperial College London, Helix Centre<br>Dickens, Daniel; Imperial College London, Helix Centre<br>Fernandez Crespo, Roberto; Imperial College London<br>Leis, Melanie; Imperial College London, Big Data and Analytical Unit, Institute of Global Health Innovation<br>Crow, Jennifer; Imperial College Healthcare NHS Trust, Hyperacute Stroke Unit<br>Taylor, Elizabeth; Kingston University and St George's University of London<br>Jones, Fiona; Kingston University and St George's University of London<br>Darzi, Ara; Imperial College London, Institute of Global Health Innovation
**Primary Subject Heading**:	Rehabilitation medicine
Secondary Subject Heading:	Neurology
Keywords:	Stroke < NEUROLOGY, REHABILITATION MEDICINE, NEUROLOGY, PUBLIC HEALTH, Telemedicine < BIOTECHNOLOGY & BIOINFORMATICS

SCHOLARONE™
Manuscripts

I, the Submitting Author has the right to grant and does grant on behalf of all authors of the Work (as defined in the below author licence), an exclusive licence and/or a non-exclusive licence for contributions from authors who are: i) UK Crown employees; ii) where BMJ has agreed a CC-BY licence shall apply, and/or iii) in accordance with the terms applicable for US Federal Government officers or employees acting as part of their official duties; on a worldwide, perpetual, irrevocable, royalty-free basis to BMJ Publishing Group Ltd ("BMJ") its licensees and where the relevant Journal is co-owned by BMJ to the co-owners of the Journal, to publish the Work in this journal and any other BMJ products and to exploit all rights, as set out in our licence.

The Submitting Author accepts and understands that any supply made under these terms is made by BMJ to the Submitting Author unless you are acting as an employee on behalf of your employer or a postgraduate student of an affiliated institution which is paying any applicable article publishing charge ("APC") for Open Access articles. Where the Submitting Author wishes to make the Work available on an Open Access basis (and intends to pay the relevant APC), the terms of reuse of such Open Access shall be governed by a Creative Commons licence – details of these licences and which Creative Commons licence will apply to this Work are set out in our licence referred to above.

Other than as permitted in any relevant BMJ Author's Self Archiving Policies, I confirm this Work has not been accepted for publication elsewhere, is not being considered for publication elsewhere and does not duplicate material already published. I confirm all authors consent to publication of this Work and authorise the granting of this licence.

For peer review only

TITLE

What is the feasibility and patient acceptability of a digital system for arm and hand rehabilitation after stroke? A mixed methods, single-arm feasibility study of the 'OnTrack' intervention for hospital and home use.

AUTHORS AND AFFILIATIONS

Gianpaolo Fusari[1], Ella Gibbs[1], Lily Hoskin[1], Anna Lawrence-Jones[1], Daniel Dickens[1], Roberto Fernandez Crespo[2], Melanie Leis[2], Jennifer Crow[3], Elizabeth Taylor[4], Fiona Jones[4], Ara Darzi[1]

[1]Helix Centre, Imperial College London and the Royal College of Art, London, United Kingdom

[2]Big Data and Analytical Unit, Institute of Global Health Innovation, Imperial College London, London, United Kingdom

[3]Stroke Unit, Charing Cross Hospital, Imperial College Healthcare NHS Trust, United Kingdom

[4]Kingston University and St George's, University of London, London, United Kingdom

Correspondence to:

Gianpaolo Fusari, Helix Centre, Imperial College London and the Royal College of Art, London, United Kingdom - gianpaolo@helixcentre.com

Word count:

4,478

KEY WORDS

Stroke, rehabilitation medicine, neurology, public health, digital health, digital therapeutics

ABSTRACT

Objectives

Arm weakness is common after stroke; repetitive activity is critical for recovery but people struggle with knowing what to do, volume, and monitoring progress. We studied the feasibility and acceptability of OnTrack, a digital intervention supporting arm and hand rehabilitation in acute and home settings.

Design

A mixed method, single-arm study evaluating the feasibility of OnTrack for hospital and home use. An independent process evaluation assessed the intervention's fidelity, dose and reach. Amendments to the protocol were necessary after Covid-19.

For peer review only - http://bmjopen.bmj.com/site/about/guidelines.xhtml

Setting

Acute stroke services and home settings in North West London.

Participants

12 adults with a stroke diagnosis <6 months previously (first or recurrent) requiring arm rehabilitation in hospital and/or home.

Intervention

12 weeks using the OnTrack system comprising arm tracking and coaching support for self-management.

Primary and secondary outcome measures

Recruitment, retention and completion rates; compliance and adherence to the intervention; reasons for study decline/withdrawal.

Intervention fidelity and acceptability, evaluated through an independent process evaluation.

Patient measures including activity baseline, healthcare activation, arm function & impairment collected at baseline, week 7 and week 14 of participation to assess suitability for a RCT.

Results

181 individuals screened, 37 met eligibility criteria, 24 recruited (65%); of these, 15 (63%) were recruited before Covid-19, and 9 (37%) during. 12 completed the intervention (50%). Despite Covid-19 disruptions, recruitment, retention and completion were in line with pre-study expectations and acceptable for a definitive trial. Participants felt the study requirements were acceptable and the intervention usable. Fidelity of delivery was acceptable according to predetermined fidelity markers. Outcome measures collected helped determine sample size estimates and primary outcomes for an RCT.

Conclusions

The intervention was found to be usable and acceptable by participants; study feasibility objectives were met and demonstrated that a definitive RCT would be viable and acceptable.

Trial registration number

NCT03944486.

STRENGTHS AND LIMITATIONS

- This was the first feasibility trial of a novel intervention employing an integrated approach for tracking arm activity and self-management coaching with the aim of increasing the opportunities for independent rehabilitation.
- Recruitment for the study began in September 2019, modifications to the protocol were necessary to enable the delivery of the intervention remotely after the start of the Covid 19 pandemic in March 2020.

For peer review only - http://bmjopen.bmj.com/site/about/guidelines.xhtml

- A Patient and Public Involvement (PPI) group met four times during the study; the group gave advice regarding modifications to the protocol, and contributed to the interpretation and dissemination of data findings.
- An independent process evaluation was carried out to provide detailed information about implementation, context, and the mechanisms of impact of the intervention.
- Longer term follow-up of participants was not possible within the timeframe set for the trial; however participant views were sought regarding the acceptability of longer-term follow-up.

INTRODUCTION

Background

Globally, five million people are left disabled from stroke, commonly with a form of arm impairment.(1) In the UK, 75% of disabled stroke survivors, or ~450,000 people, have an arm weakness.(2) By 2035, this number may increase by a third.(3) Stroke costs the UK society £26b annually, by 2025 costs could increase to £43b and to £75b by 2035. An ageing population, better stroke survival rates, and the overall increase in labour costs account for this trend.(3)

Understanding when to provide interventions that can improve arm function is recognised as a national research priority;(4) despite this, time spent providing therapy for the arm is often limited, resulting in patients spending minimal time rehabilitating or being active.(5,6) There is a correlation between physical activity and the ability to perform activities of daily living using the arm,(7) but a Cochrane review of over 500 trials failed to yield high-quality practice recommendations for arm interventions.(8) The Covid-19 pandemic exacerbated this problem resulting in thousands of stroke survivors receiving diminished rehabilitation and an increase in health inequalities.(9,10) Without specialist support, rehabilitation can be less effective and an isolating experience.(11)

Ethnographic studies describe how patients struggle to see and keep track of improvements(12-14) - especially outside of scheduled one-to-one therapy - having an impact on motivation and creating dependency on therapists for feedback.(6,7,15,16) Stroke survivors often report feeling unsupported after leaving hospital and not knowing how to best help themselves improve their arm function.

We believe repetitive activity can be increased by targeting the time patients go about their daily activities and could use their arm movement (however small) to a greater extent. Capacity for activity could be increased further by using self-management strategies as demonstrated by several programmes in stroke and other long-term conditions.(17-20) Additionally, there is strong evidence to suggest that digital interventions (21), and in particular digital therapeutics (DTx), (22) could help support stroke patients in their rehabilitation and life after stroke.

The aim of this study was to assess the feasibility of the OnTrack intervention - which could increase opportunities for arm activity by improving individuals' self-management skills through tailored support and real-time activity feedback - and inform the design of a definitive randomised controlled trial (RCT).

As per study protocol,(23) recruitment, adherence and retention rates were measured, as well as reasons for declining participation or withdrawing from the trial; the utility and completion of outcome measures to provide an indication of effectiveness to inform sample size calculations for an RCT; the usability of OnTrack by study participants; and the acceptability of study procedures by front-line staff. An independent process evaluation assessed the fidelity of the intervention delivery as well as the acceptability of the intervention to participants and delivery team.

For peer review only - http://bmjopen.bmj.com/site/about/guidelines.xhtml

METHODS

Design

A feasibility study with a nested process evaluation. The study was a single-site, non-randomised intervention trial. The design of the study was developed through a collaborative approach between the authors, a PPI steering group, stroke therapists (occupational therapists and physiotherapists) and the Research Design Service at the National Institute for Health Research (NIHR).

Study settings

Prior to lockdown restrictions caused by the Covid-19 pandemic, participants were enrolled in the study at an inner city NHS hospital Trust in West London and, where necessary, continued their participation at home after discharge to complete the 14-week study period. After restrictions were introduced, all participants were enrolled at home and were followed remotely for 14 weeks.

Participants

Participant inclusion and exclusion criteria are fully defined in the study protocol (23); briefly, participants were adults with a stroke diagnosis less than 6 months previously (first or recurrent) requiring arm and hand rehabilitation in hospital and/or at home, medically stable, presenting without arm pain or oedema, and with capacity to consent.

Recruitment

Participants were recruited from the Hyperacute Stroke Unit (HASU), Acute Stroke Unit (ASU) and Clinical Neuro Rehabilitation Unit (CNRU) at an NHS Hospital Trust in London.

Recruitment followed two distinct procedures before and after lockdown restrictions were in place. Between August 2019 and March 2020, hospital therapy teams were responsible for screening, introducing the study, and providing information documents to potential participants. After taking consent, therapists shared patient information with the research team. Recruitment was suspended between March and August 2020. From September 2020 onwards, therapy teams continued to perform eligibility screens sharing patient information with the research team only at discharge. The consent and recruitment process was then completed remotely at participants' homes.

Intervention

Participants were enrolled in the study for 14 weeks. Week 1 consisted of baseline assessments; weeks 2-13 consisted of the OnTrack intervention (detailed below); week 14 consisted of follow up assessments and an interview performed by the process evaluation team. Additionally, a mid-participation assessment was performed during week 8.

The intervention was the OnTrack system, consisting of three main components:

1. Activity tracking. All participants were asked to wear a smartwatch on their affected arm during waking hours (typically 12 hours) which included software and a purpose-built algorithm providing data on gross arm activity (minutes of activity performed).

2. Motivational content. Delivered as visual feedback on the amount of activity performed, personalised in-app messages, and links to educational material. A sample of content can be seen in Table 1.

For peer review only - http://bmjopen.bmj.com/site/about/guidelines.xhtml

3. Self-management coaching. Delivered during 1-1 coaching sessions (in person and remotely). The coaching component has been influenced by the self-management principles as defined by the Bridges Self-Management Programme (24) and the Taking Charge After Stroke (25) self-management programme. Details of the coaching component are provided as a supplementary file.

Activity data collected by participants was monitored remotely by the research team, this helped to guide conversations during coaching sessions.

The coaching component was reviewed at the study's halfway point following data analysis performed by the process evaluation team in discussion with the PPI group.

Participants were loaned all equipment necessary for the trial and no previous experience with using smart devices was required to participate. Technical support was provided by the intervention team in cases where the hardware and/or software failed to perform the required functions to deliver the intervention.

Table 1 - In-app messages

Messages and links to content related to stroke rehabilitation and self-management were sent to participants via the OnTrack smartphone application. Messages were divided into four different categories.

Message category	Description	Frequency, time of day	Sample message
Intention settings	Message to set the participant's intentions for the day. Participants were able to respond to these messages with a Yes / No answer.	Weekdays, in the morning	"Good morning Jon!<br><br>Aim to wear the watch on your LEFT wrist for as long as you can today and keep an eye on your arm activity target of 45 minutes.<br><br>Are you ready to try this?"
Tips & advice	Personalised messages to include tips or advice relevant to the participant's situation. This type of message did not require a response.	2-3 times per week, at midday	"Weekends are often different from the rest of the week but you can still include activities that involve your LEFT arm.<br><br>It's also ok to have a rest but don't forget to use appropriate cutlery for every meal if you have the opportunity as we discussed before.<br><br>You've got this!"
Reflective practice	Message with the intention to help participants reflect on their progress. This type of message did not require a response.	2-3 times per week, towards the end of the day	"Hi Jon!<br><br>I hope you managed to reach your target of 45 minutes of arm activity today.<br><br>Were you able to think about new ways of involving your arm?<br><br>Let's talk about these when we meet!"
Links to external content	Participants in Phase 1 of the study received a total of 9 links whilst participants in Phase 2 received 11 links signposting to resources they could tap into once their	9-11 links sent over 14 days, at midday	"Hi Jon, the Stroke Foundation in Australia have put together a great blog post on how to improve your Problem Solving skills.

For peer review only - http://bmjopen.bmj.com/site/about/guidelines.xhtml

	participation ended.		Click here to check it out when you get a chance!" (link)

Outcomes

Feasibility of trial design and procedures

Outcomes included the measurement of recruitment rates, including number of patients screened, eligible, consented, and excluded after screening; participant adherence to the intervention and usage (percentage of days using OnTrack, minutes of activity as recorded using OnTrack, engagement with features in the OnTrack app); completion rates; and acceptability and reasons for decline/withdrawal.

NHS therapists responsible for screening and recruitment were invited to complete a survey to gather their feedback regarding acceptability of study procedures.

Clinical assessments

Clinical outcomes were collected to identify an appropriate primary outcome, and to estimate parameters for a sample size calculation for an RCT, these were collected at the start, halfway and end of participation (Table 2).

Table 2 - Outcome measure schedule

Concept	Assessment	Week performed
Patient Activation / Engagement	Patient Activation Measure (PAM)(26,27)	1, 8*, 14
Arm impairment	Fugl-Meyer Assessment for upper extremity (FMA-UE)**(28,29)	1, 8*, 14
Arm function	Upper-Extremity Motor Activity Log-14 (MAL)(30)	1, 8*, 14
Gross level of disability	modified Rankin Scale (mRS)(31)	1, 8*, 14
Arm pain	Visual Analogue Scale (VAS)(32)	1, 8*, 14
Cognitive impairment	Montreal Cognitive Assessment (MoCA)†(33,34)	1, 8*, 14
Arm neglect	Albert's Test (AT)(35)	1, 8*, 14
Quality of life	EQ-5D-5L(36)	1, 8*, 14
Activity baseline	Axivity AX3 usage on both arms	1, 14
System usability	System Usability Scale (SUS)(37)	14

* Performed at week 7 during Phase 2 of the study
** Not performed during Phase 2 due to inability to administer remotely
† A modified version was performed during Phase 2

Additional assessments

Activity baselines were gathered at weeks 1 and 14 of participation, study participants wore activity trackers (Axivity AX3) on both arms during waking hours (typically 12 hours/day) for 3-7 days to gather activity baseline data and allowing for left-right arm usage comparison.

For peer review only - http://bmjopen.bmj.com/site/about/guidelines.xhtml

The System Usability Scale (SUS)(37) was used to subjectively assess the usability of the OnTrack intervention.

Process evaluation

An independent process evaluation was conducted in parallel to this trial. The evaluation aimed to determine whether the intervention was delivered as intended and to understand the mechanisms of impact. Interviews were conducted with participants finishing the intervention period. These focused on their experience of using OnTrack and the different components of the intervention, as well as on their perceptions of the impact OnTrack had on their rehabilitation and ability to self-manage.

Sample size

The sample size was determined using guidelines that advocate a sample size between 12-30 for feasibility studies.(38) We aimed to recruit a minimum of 24 participants over the duration of the study which was in line with referral rates at the recruiting site.

Analysis

Analysis was performed on the study parameters and its implementation. We evaluated the usage of OnTrack as well as outcome measures and recorded changes over time.

Patterns of activity were analysed by day and hour of day. Comparisons between activity data and usage of different application features were created to understand their influence in activity output. Similarly, usage of individual features were recorded (e.g. engagement with messages, number of times activity stats were accessed, etc.). Furthermore, a comparison between activity minutes and performance in the different outcome measures was performed.

Pearson correlation coefficient was used to explore relationships between application usage and patient activity. A multivariate logistic regression model was used to investigate the independent effects of the the daily activity time of each patient, measured in 30 minute intervals ('Activity'), the number of days since the patient enrolment in the trial ('Time'), and whether the patient suffered a stroke on their dominant arm ('Dominant arm') on the patients' left-right arm usage ratio. Multivariate linear regression models were used to assess the independent effects of the 'Activity' and 'Time' variables on the other outcome measures in this study.

Data collected for the process evaluation was a combination of qualitative data from semi-structured interviews and observations of coaching sessions. At the study half-point, a workshop was conducted with the PPI group who analysed interview transcripts and made recommendations to refine the protocol.

Patient and public involvement (PPI)

A PPI group comprising three stroke survivors helped refine the intervention to the version used for this study. The group met a total of 4 times over the duration of the study, their time and travel were reimbursed according to INVOLVE (39) guidelines. The group supervised the development of all patient-facing material to ensure its clarity and accessibility. Members were trained by experienced researchers to participate in qualitative data analysis at the study's halfway point. They helped to refine themes and key messages arising from qualitative interviews. The PPI involvement plan was shared with Imperial College London's PPI 'Research Partners Group' to ensure the needs of the steering group were accounted for.

For peer review only - http://bmjopen.bmj.com/site/about/guidelines.xhtml

RESULTS

Recruitment

Recruitment took place between August 2019 and December 2020 with an imposed study suspension between March 2020 and August 2020 due to the public health emergency caused by the Covid-19 pandemic.

In total, 181 individuals were screened for eligibility with 37 meeting the inclusion criteria invited to participate (Figure 1). 24 participants were recruited with a response rate of 65%, of these, 15 (63%) were recruited before the study suspension and 9 (37%) between September and December 2020.

Participant characteristics

Data collected from participants during the initial assessment was available for 21 participants (11 females; mean age 61.1, range 33.5 - 82.5), three participants were lost to follow up before a session could be arranged. For the majority of participants (n=16, 76%) this was their first stroke, only one participant had had more than two previous strokes. Onset of the last stroke varied between 11 and 141 days with a mean of 42.2 days. 7 participants (33%) were impaired on their dominant arm. All participants owned or had experience with mobile phones but only 15 (71%) were smartphone users; 18 (86%) had WiFi at home. Although this was not a randomised study, participants' acceptance of randomisation was queried; 13 (62%) expressed they would have consented to the study regardless of whether they'd be allocated to a control or intervention group.

All participants underwent some form of rehabilitation treatment by a UK NHS acute and/or community provider, ranging from in-person ESD to remote community neurorehabilitation. However, the study did not collect detailed information on the type of rehabilitation that was provided.

Participant baseline characteristics can be seen on Table 3.

**Table 3 - Participant characteristics at baseline (n=21)**	
Gender	11 female (52%)
Age (years); mean (SD); median (min, max)	61.1 (12.5); 60.5 (33.5 min, 82.5 max)
Ethnicity; n (%)	
White British	7 (33%)
White other	3 (14%)
Asian	5 (25%)
Black	3 (14%)
Other	2 (10%)
Prefer not to say	1 (5%)
Impaired arm	7 Right (33%)
Dominant arm	19 Right (90%)
Dominant arm impaired	7 Yes (33%)
Stroke onset (days); mean (SD); median (min, max)	42.2 (33.04); 33 (11 min, 141 max)

For peer review only - http://bmjopen.bmj.com/site/about/guidelines.xhtml

Table 3 - Participant characteristics at baseline (n=21)	
Gender	11 female (52%)
Type of stroke; n (%)	
Ischaemic	11 (52%)
Hemorrhagic	2 (10%)
Unknown	8 (38%)
First stroke	16 Yes (76%)
Comorbidities	15 Yes (71%)
Smoker	5 Yes (24%)
Smartphone user	15 Yes (71%)
WiFi at home	18 Yes (86%)
Amenable to randomisation	13 Yes (62%), 2 No (10%), 6 Don't know (29%)
SD = standard deviation	

Adherence and retention

Of the 24 participants recruited, 21 (87%) took part in an initial session with researchers to gather baseline characteristics; 18 (75%) completed outcome measures and 17 (71%) went through to complete the activity baseline week. Of the 7 participants dropping out at this stage, researchers were unable to make contact with 5 of them; 1 was readmitted to hospital; and 1 decided to stop participation after feeling numbness on the arm.

17 (71%) participants started using OnTrack; 14 (58%) repeated outcome measures at halfway (week 7-8 of participation); and 12 (50%) completed the intervention period, repeated outcome measures, and completed the final activity baseline week. Of the 5 participants lost between starting OnTrack and finishing the study, 3 withdrew consent and 2 were lost to follow up. Reasons for withdrawing consent included one person who felt they had gained all they could from OnTrack and wanted to continue on their own; another felt that the intervention would not help them as they had too much on their mind; and another felt too anxious, fatigued and had a skin condition that was worsening.

From the 12 participants completing the intervention, one was unable to attend the final interview as they finished participation the week lockdown started in the UK and were living in sheltered accommodation making it impossible for the process evaluation team to make contact.

Adverse events

No serious adverse events were reported for this study. One participant was admitted to hospital at week 8 of participation for reasons unrelated to the study. Their participation was suspended for 4 weeks and later resumed in agreement between the participant, their family and the researchers.

On two occasions, participants reported feeling unwell and took one or two days off the intervention. They did not record their activity and no messages were sent to them during this period.

For peer review only - http://bmjopen.bmj.com/site/about/guidelines.xhtml

Two instances of data loss occurred, 5 weeks of data were lost for one participant and one week for another. Failure to sync data between the local devices (watch and smartphone) and the server accounted for these instances. This did not have an effect on the experience for the participants as they were able to access their data locally on their devices.

Acceptability and usability of OnTrack

Activity tracking

Compliance rate with activity tracking was measured by dividing the total number of days on the intervention by the total number of days the participant recorded their arm activity on the smartwatch. On average, participants were on the intervention for 83 days (min 76, max 84) and recorded their activity on 71 days (min 47, max 84), or the equivalent of 6.1 days per week for a compliance rate of 86% (Figure 2).

Furthermore an indication of usability and engagement with the intervention could be gathered by measuring the number of daily data interactions participants had using the applications on the smartphone and watch. Figure 3 shows how participants kept track of their activity data on the watch by looking at their activity data a mean of 7.5 times per day (min 4.5, max 12.3) and 3.5 times daily on the smartphone application (min 1, max 9.4).

A 63% mean increase in activity (min -1%, max 864%) could be observed in all participants when comparing the start and end of participation - or the equivalent of 4.3 more hours of activity per week. Participants partially attributed their motivation to do more activity to having a daily target of minutes to achieve. On average, participants increased their target by 5% every session and managed an overall increase of 75% (min 18%, max 200%) between weeks 1 and 12, reaching their target on 3.1 days every week (Figure 4).

Messages and educational content

We measured the way participants engaged with messages, their views were explored during interviews. One participant was deemed cognitively unable to engage with messaging content, this participant was excluded from the messaging component of the intervention.

Overall, an average of 1.5 messages were sent per day and they were opened 1.3 times per day (Figure 5). Engagement with messages was lower than expected. One possible explanation is that messages were only sent to the smartphone and no notifications were received on the watch, if the participant didn't have the phone with them they would miss the message notification. In addition most participants felt that carrying the phone was sometimes a burden and so the OnTrack phone was left behind for long portions of the day.

Correlation between measured activity and application analytics

Correlations between recorded patient activity and application usage analytics were calculated in order to determine if these variables could be of interest for future analysis. There was a significant correlation between patients' activity and their daily activity target ($p < 0.001$, $R = 0.164$), however this correlation did not appear to be significant when compared to the weekly activity target ($p = 0.323$, $R=-0.035$). There was no significant association with the number of times patients saw their activity graph on their watch ($R = -0.035$, $p=0.323$). We did not identify a significant difference (Wilcoxon Signed Ranks Test (40), $p=0.28$) in activity minutes based on whether or not patients accepted their intention for the day (Yes = 91.7 minutes, No answer = 64.4 minutes). Finally, there were positive and significant correlations with the number of times patients viewed messages sent ($p < 0.001$, $R = 0.201$), and the number of links opened ($p = 0.002$, $R = 0.107$).

For peer review only - http://bmjopen.bmj.com/site/about/guidelines.xhtml

Views on the intervention and study procedures

Participant views

Participants provided feedback on the study procedures, they felt that recruitment procedures were acceptable and that the information they received was appropriate. Some participants felt that the amount of assessments conducted could be tiring and recommended either reducing them or conducting them over two or more sessions. The activity sensors worn on both wrists during the first and last weeks of participation were uncomfortable for some participants and some required help to put them on.

An in-depth analysis on the experience of participating and using the intervention is reported in a process evaluation publication. In summary, participants felt OnTrack was working as expected and intended. They expressed feeling well supported to use the technology, even without prior experience. They reported feeling that the activity targets were motivating, although they would welcome more specific activity recommendations alongside targets. All participants mentioned that the quality of the coaching was an important part of creating a positive experience. Participants found remote sessions acceptable and reported valuing the contact during lockdown.

Therapist views

Therapists contributing to screening and recruitment completed an online survey exploring three themes: the time spent performing study procedures; the intervention and the potential benefits to stroke survivors; and views on patient engagement needed to use OnTrack. 6 of 13 therapists completed the survey (46.2% completion rate).

Overall, therapists felt that the time they spent in study procedures was in line with expectations, and that the tasks performed were acceptable.

On The potential benefits of the intervention, therapists thought that the intervention could be beneficial in general terms and that it could be of most benefit to stroke survivors with motor and sensory impairment. Benefits to patients with arm neglect were less clear but tended to the positive.

Therapists agreed that the intervention could help motivate patients into performing more activities with their impaired arm and support them in the self-management of their recovery. However, some respondents felt that patients who are less engaged with their recovery may not fully benefit from the intervention.

Clinical outcomes

A wide range of outcome measures were collected (Table 4) in order to assess their suitability and feasibility for use during a future definitive study. Pandemic restrictions brought forward new considerations; for example, despite being a widely used assessment for arm impairment after stroke, the FMA-UE was only used during Phase 1 of the study as it was impossible to perform once the study moved to a remote format of delivery. On the other hand, the MAL can be performed remotely and has proven to be a reliable and valid measure of outcome from rehabilitation and of functional status in patients with arm impairment post stroke.(30) An estimate sample size of 46 was calculated using the MAL's reported minimal clinically important difference (41) with 90% power and two-sided alpha of 5%.

A member of the research team was responsible for completing all assessments either in person (Phase 1) or remotely (Phase 2). Outcomes are reported for the 12 participants completing the intervention.

For peer review only - http://bmjopen.bmj.com/site/about/guidelines.xhtml

Table 4 - Outcome measures at baseline and follow-up points, mean and (SD)

Outcome	Baseline	Halfway*	14 weeks
PAM	69.7 (17.8)	65.8 (14.8)	68.1 (10.3)
FMA-UE**	37.7 (17.2)	39.0 (20.1)	36.4 (22.4)
MAL***	2.00 (1.4) | 2.26 (1.5)	2.94 (1.2) | 3.09 (1.2)	3.24 (1.3) | 3.17 (1.2)
mRS	2.8 (1.1)	2.5 (0.8)	1.9 (0.9)
VAS (pain)	0.8 (1.0)	2.5 (1.9)	2.8 (2.3)
MoCA†	22.6 (6.9)	24.0 (5.1)	25.3 (1.0)
MoCA††	17.8 (3.4)	19.0 (1.9)	18.3 (2.4)
AT	0.2 (0.4)	0.1 (0.3)	0.1 (0.3)
EQ-5D-5L index	0.462 (0.3)	0.585 (0.1)	0.606 (0.2)
EQ-5D-5L VAS	57.3 (20.7)	72.7 (9.8)	74.2 (15.1)
SUS			84.6 (13.1)

*8 weeks for Phase 1 participants; 7 weeks for Phase 2.
** Only performed during Phase 1. Participants who started in Phase 1 but finished their participation after lock down, did not complete this measure subsequently.
*** How much score | How well score
† Full version applied before lockdown measures (scores out of 30)
†† Telephone version applied after lockdown measures (scores out of 22)
PAM - Patient Activation Measure; FMA-UE - FuglMeyer Upper Extremity; MAL - Motor Activity Log; mRS - Modified Rankin Scale; VAS - Visual Analogue Scale (pain); MoCA - Montreal Cognitive Assessment; AT - Albert's Test; EQ-5D-5L - EuroQol; SUS - System Usability Scale.

Correlations between measured activity and outcome measures

Several outcome variables were modelled (Table 5). Individual models were created for each of the following variables: left-right arm usage ratio improvement, which measures whether or not the patients' difference in arm usage improved by the end of the study (this model also included an additional independent variable which captured whether or not the patients' impaired arm was their dominant arm, model fit p < 0.001), PAM (model fit p = 0.002), VAS (model fit p < 0.001), MAL - How much score (model fit p < 0.001), MAL - how well score (model fit p < 0.001), mRS (model fit p < 0.001), MoCA (model fit p = 0.158), EQ-5D-5L index (model fit p < 0.001), EQ-5D-5L VAS (model fit p < 0.001).

Table 5 - Outcome measures model estimates

Models were created by using the outcome variable as a dependent variable, and using the variables listed under the 'Model variables' as covariates. 'Activity' quantifies segments of 30-minutes of daily activity, 'Time' determines the number of days since each patient started recording their activity, and 'Dominant arm' was used to determine whether or not the patient suffered a stroke on their dominant arm.

Outcome	Model adjusted R^2	Model fit p value	Model variables	Estimates	Estimate p value
Left-Right ratio improvement* (Ref. category: No improvement)	0.169†	< 0.001	Activity<br>Time<br>Dominant arm (Ref. category: No)	1.005††<br>0.995††<br>6.684††	< 0.001<br>0.116<br>< 0.001

For peer review only - http://bmjopen.bmj.com/site/about/guidelines.xhtml

PAM	0.012	0.002	Activity<br>Time	-0.653<br>-0.022	0.002<br>0.290
VAS	0.135	< 0.001	Activity<br>Time	-0.056<br>0.29	0.025<br>0.000
MAL - how much	0.452	< 0.001	Activity<br>Time	0.304<br>0.012	< 0.001<br>< 0.001
MAL- how well	0.433	< 0.001	Activity<br>Time	0.324<br>0.001	< 0.001<br>< 0.001
mRS	0.267	< 0.001	Activity<br>Time	-0.161<br>-0.007	< 0.001<br>< 0.001
MoCA	0.002	0.158	Activity<br>Time	0.002<br>0.000	0.272<br>0.160
EQ-5D-5L index	< 0.001	0.161	Activity<br>Time	0.020<br>0.002	< 0.001<br>< 0.001
EQ-5D-5L VAS	0.226	<0.001	Activity<br>Time	1.252<br>0.241	< 0.001<br>< 0.001

* Left-right ratio improvement was measured using a logistic regression model, as opposed to other variables which were modeled using a linear model.
† Pseudo R^2 (McFadden)
†† Estimated for the left-right ratio improvement are show as add ratios

Fidelity of intervention delivery

Fidelity of delivery was assessed through the observation of coaching sessions. Observations during the first half of the study identified that the delivery of the coaching sessions was inconsistent with how participants were experiencing their recovery. This resulted in the intervention team consistently deviating from the coaching plan in response to participant needs.

A workshop involving the PPI group was planned at the study half-way point to refine the delivery of the intervention for Phase 2. A summary of findings from the half-way workshop suggested that overall, the OnTrack intervention was working as intended, however the following points were observed:

- Participants reported a lack of clarity about whether the focus of the intervention was to improve hand and arm movement, or to improve activity more generally.
- In coaching sessions, the discussions relating to the activity target were distinct from those relating to individual functional goals, and the self-management strategies were not easy to observe.
- The activity targets were regarded as motivating.
- Participants wanted more specific activities as targets, e.g. holding a cup, making toast.
- Participants valued facts and useful information in messages.
- The coaches were seen as central – the language and strategies they use is key to promoting self-management.

In response to these findings, a more fluid and flexible approach to coaching was developed. The new guide included prompts for supporting self-management during all sessions, as opposed to being session specific, it encouraged focusing more on discussing the meaningful activities that participants would like to use their arms for, and using the activity data to support this (a table describing these changes is provided as a supplementary file).

Delivery of the intervention during Phase 2 was affected by the coronavirus pandemic. All sessions were delivered remotely, participants stated that this was acceptable and welcomed the contact with coaches during lockdown.

For peer review only - http://bmjopen.bmj.com/site/about/guidelines.xhtml

DISCUSSION

Summary of principal findings

This study aimed to assess the acceptability of a novel digital intervention to support arm and hand rehabilitation after stroke and the feasibility of conducting a definitive trial into its effectiveness. The study met its objectives in assessing the acceptability and feasibility of the intervention and trial procedures. Despite being affected by the pandemic, recruitment to the trial was in line with pre-study expectations as described in the protocol's sample size calculation (23), as well as participant retention rates. Appropriate outcome measures and sample size estimations for an RCT were analysed. Qualitative data were gathered showing that the intervention was usable and acceptable to study participants, but most importantly, it highlighted the components of OnTrack that were perceived as most valuable to them.

Strengths and weaknesses of the study

Findings from this study have shown a positive way in which wearable technology and self-management coaching could be combined to improve rehabilitation outcomes by helping participants identify opportunities for self-practice and become more engaged in their recovery. Previous studies involving wearable technologies to encourage more practice at home have shown positive results (22, 42); similarly self-management approaches have been shown to obtain better outcomes for stroke patients (17-20). To our knowledge, this is the first study aiming to combine these two approaches to provide a viable intervention that can be delivered in conjunction with usual care.

The coronavirus pandemic significantly affected the running of this study. In the first instance, a suspension in recruitment was necessary, followed by an amendment to the recruitment and consenting procedures. In addition, the adaptation to lockdown restrictions accelerated the need for remote delivery of OnTrack, this however had little impact on participants' experiences. As explored in the context of digital therapeutics (22), this may represent an opportunity to challenge conventional clinical practice and may bring a more direct access to services for patients. Furthermore, it may bring additional benefits such as closer monitoring at a lower cost and optimisation of clinician/patient interactions. Future studies should help provide answers to these questions.

The independent process evaluation provided an opportunity to understand the different mechanisms of impact. Allowing for refinement at the half-way point enabled the researchers to incorporate feedback to modify the way OnTrack was delivered. The engagement of the PPI group enabled this refinement to be implemented through a user-centred process. However, the process evaluation also found that the messaging component of the intervention received mixed reviews by participants. On the one hand, the practical information sent was perceived as valuable, but the tone of voice and frequency of other messages was questioned by some participants. This should be revised for a future version of OnTrack.

Due to pragmatic reasons, the study could not cater for longer term participant follow-up. During interviews, participants expressed they would value a check some time after the program ended. A future study should consider incorporating longer term follow-up to understand if participants continue to apply self-management principles.

Unanswered questions and future research

Whilst randomisation was not possible during this study, participants were asked if they would be willing to participate in a randomised study. Most participants said that randomisation would not influence their decision to participate, however more consideration is needed to understand the appropriate design of control and intervention arms.

For peer review only - http://bmjopen.bmj.com/site/about/guidelines.xhtml

The messaging component of this intervention needs further refinement and co-design in collaboration with stroke survivors. Whilst, for the most part, the messages are seen as a valuable component, their design requires a better understanding of the content, context, frequency and tone of voice in which they are delivered.

The intervention was delivered over 12 weeks (plus 2 weeks of assessments at each end), however questions remain as to whether OnTrack could be delivered over shorter or longer periods and how this would affect the design of the coaching component. The coaching component itself requires further thinking in how it may be delivered at scale, the training required, and other tools needed to support this role. For this trial, coaching guidelines were used and coaches had access to a digital dashboard showing participants' activity. Future development and studies should focus on understanding these components from the perspective of therapy teams. In addition to this, it will also be necessary to provide answers regarding the economic impact that the intervention may have when delivered as an adjunct to standard care in a national health service such as the UK NHS.

Conclusion

This study aimed to assess the feasibility, acceptability and safety of the OnTrack intervention when used to support arm and hand rehabilitation in acute and home environments. The results obtained through this study indicated that participants found the intervention to be safe, usable and acceptable. Moreover, the study's feasibility objectives were met and provide a basis to support further investigation to demonstrate the intervention's clinical effectiveness through a randomised control trial.

ORIGINAL PROTOCOL

https://bmjopen.bmj.com/content/10/3/e034936

AUTHOR CONTRIBUTIONS

AD was Principal Investigator and grant holder and oversaw the project along with DD. GF, EG and LH developed the intervention and conceived of the study. GF, EG, and FJ initiated the study design and ET and ML helped with further refinement. ALJ led on PPI. EG and GF were responsible for delivering the intervention and data collection. JC had participant screening oversight. FJ and ET were responsible for the process evaluation. ML and RFC conducted the primary statistical analysis. All authors contributed to this manuscript and approved the final version.

FUNDING STATEMENT

This study was funded by the NIHR Imperial Biomedical Research Centre (BRC), grant 1215-20013. The views expressed are those of the author(s) and not necessarily those of the NIHR or the Department of Health and Social Care.

DATA SHARING STATEMENT

All data relevant to the study are included in the article or uploaded as supplementary information. Additional data relevant to the study are available on reasonable request and should be directed to the corresponding author.

For peer review only - http://bmjopen.bmj.com/site/about/guidelines.xhtml

CONFLICT OF INTERESTS

None.

ACKNOWLEDGMENTS

The authors would like to acknowledge the contributions of all the members of the PPI group; all participants involved; Peter Wilding, Meenakshi Nayar, and Davina Richardson and therapy teams at ICHT; therapy teams at CLCH; Ruth Nicholson for her support in ethics applications and procedures; Steve McAteer and Eleni Daniels for their guidance on the BRC program; Dr Hendramoorthy Maheswaran for his help reviewing the manuscript.

ETHICS STATEMENT

Approval granted by the NHS Health Research Authority, Health and Care Research Wales, and the London—Surrey Research Ethics Committee (ref. 19/LO/0881).

REFERENCES

(1) WHO. *The Atlas of Heart Disease and Stroke*. Available from: https://www.who.int/cardiovascular_diseases/resources/atlas/en/ [Accessed 04 Oct 2019].

(2) Stroke Association. *State of the Nation: stroke statistics*. Stroke Association. 2018.

(3) Patel, A., Berdunov, V., King, D., Quayyum, Z., Wittenberg, R. & Knapp, M. *Current, future and avoidable costs of stroke in the UK* . Stroke Association. 2017.

(4) *Shaping stroke research to rebuild lives: The Stroke Priority Setting Partnership results for investment.* 2021.

(5) Care Quality Commission. *Supporting life after stroke: a review of services for people who have had a stroke and their carers*. London: Care Quality Commission. 2011.

(6) Bernhardt J, Dewey H, Thrift A, Donnan G. Inactive and Alone: Physical Activity Within the First 14 Days of Acute Stroke Unit Care. *Stroke*. 2004; 35 (4): 1005-1009. Available from: doi: 10.1161/01.STR.0000120727.40792.40 Available from: http://stroke.ahajournals.org/cgi/content/abstract/35/4/1005 .

(7) Kunkel D, Fitton C, Burnett M, Ashburn A. Physical inactivity post-stroke: a 3-year longitudinal study. *Disability & Rehabilitation*. 2015; 37 (4): 304-310. Available from: doi: 10.3109/09638288.2014.918190 Available from: http://www.tandfonline.com/doi/abs/10.3109/09638288.2014.918190 .

(8) Pollock A, Farmer SE, Brady MC, Langhorne P, Mead GE, Mehrholz J, et al. Interventions for improving upper limb function after stroke. *The Cochrane database of systematic reviews*. 2014; 2014 (11): CD010820. Available from: doi: 10.1002/14651858.CD010820.pub2 Available from: https://www.ncbi.nlm.nih.gov/pubmed/25387001 .

(9) Royal College of Physicians. *Clinical guide for the management of stroke patients during the coronavirus pandemic*. 2020.

(10) *Coronavirus and stroke (Stroke Association)*. Available from: https://www.stroke.org.uk/finding-support/coronavirus-covid-19-and-stroke.

(11) Salter K, Hellings C, Foley N, Teasell R. The Experience of Living with Stroke: A Qualitative Meta-Synthesis. *Journal of Rehabilitation Medicine*. 2008; 40 (8): 595-602. Available from: doi:

For peer review only - http://bmjopen.bmj.com/site/about/guidelines.xhtml

10.2340/16501977-0238 Available from: http://www.ingentaconnect.com/content/mjl/sreh/2008/00000040/00000008/art00003 .

(12) Buckland D. Leaving hospital is 'like falling off a cliff' for stroke survivors. *Raconteur.* APRIL 27, 2017 . Available from: https://www.raconteur.net/healthcare/leaving-hospital-is-like-falling-off-a-cliff-for-stroke-survivors [Accessed Oct 04 2019].

(13) McKevitt C, Fudge N, Redfern J, Sheldenkar A, Crichton S, Rudd AR, et al. Self-Reported Long-Term Needs After Stroke. *Stroke.* 2011; 42 (5): 1398-1403. Available from: doi: 10.1161/STROKEAHA.110.598839 Available from: https://www.ncbi.nlm.nih.gov/pubmed/21441153

(14) Woodman P, Riazi A, Pereira C, Jones F. Social participation post stroke: a meta-ethnographic review of the experiences and views of community-dwelling stroke survivors. *Disability & Rehabilitation.* 2014; 36 (24): 2031-2043. Available from: doi: 10.3109/09638288.2014.887796 Available from: http://www.tandfonline.com/doi/abs/10.3109/09638288.2014.887796 .

(15) Jones F, Gombert K, Honey S, Cloud G, Harris R, Macdonald A, et al. Addressing inactivity after stroke: The Collaborative Rehabilitation in Acute Stroke (CREATE) study. *International journal of stroke.* 2021; 16 (6): 669-682. Available from: doi: 10.1177/1747493020969367 Available from: https://journals.sagepub.com/doi/full/10.1177/1747493020969367 .

(16) Esmonde T, McGinley J, Wittwer J, Goldie P, Martin C. Stroke rehabilitation: patient activity during non-therapy time. *Australian Journal of Physiotherapy.* 1997; 43 (1): 43-51. Available from: doi: 10.1016/S0004-9514(14)60401-3 Available from: https://www.sciencedirect.com/science/article/pii/S0004951414604013 .

(17) Jones F, Gage H, Drummond A, Bhalla A, Grant R, Lennon S, et al. Feasibility study of an integrated stroke self-management programme: a cluster-randomised controlled trial. *BMJ Open.* 2016; 6 (1): e008900. Available from: doi: 10.1136/bmjopen-2015-008900 Available from: http://dx.doi.org/10.1136/bmjopen-2015-008900 .

(18) Harwood M, Weatherall M, Talemaitoga A, Barber PA, Gommans J, Taylor W, et al. Taking charge after stroke: promoting self-directed rehabilitation to improve quality of life – a randomized controlled trial. *Clinical Rehabilitation.* 2012; 26 (6): 493-501. Available from: doi: 10.1177/0269215511426017 Available from: https://journals.sagepub.com/doi/full/10.1177/0269215511426017 .

(19) Kendall E, Catalano T, Kuipers P, Posner N, Buys N, Charker J. Recovery following stroke: The role of self-management education. *Social Science & Medicine.* 2007; 64 (3): 735-746. Available from: doi: 10.1016/j.socscimed.2006.09.012 Available from: https://www.sciencedirect.com/science/article/pii/S0277953606004801 .

(20) Grady PA, Gough LL. Self-Management: A Comprehensive Approach to Management of Chronic Conditions. *American journal of public health.* 2014; 104 (8): e25-e31. Available from: doi: 10.2105/AJPH.2014.302041 Available from: https://www.ncbi.nlm.nih.gov/pubmed/24922170 .

(21) WHO guideline Recommendations on Digital Interventions for Health System Strengthening. Geneva: World Health Organization; 2019. 1, Introduction. Available from: https://www.ncbi.nlm.nih.gov/books/NBK541905/

(22) Abbadessa G, Brigo F, Clerico M, De Mercanti S, Trojsi F, Tedeschi G, et al. Digital therapeutics in neurology. Journal of neurology. 2021; 269 (3): 1209-1224. Available from: doi: 10.1007/s00415-021-10608-4 Available from: https://link.springer.com/article/10.1007/s00415-021-10608-4 .

(23) Fusari G, Gibbs E, Hoskin L, et al. Protocol for a feasibility study of OnTrack: a digital system for upper limb rehabilitation after stroke. BMJ Open 2020;10:e034936. doi:10.1136/ bmjopen-2019-034936

(24) Bridges Self-Management Limited. *Bridges Self-Management.* Available from: http://www.bridgesselfmanagement.org.uk/ [Accessed 07 Oct 2019].

For peer review only - http://bmjopen.bmj.com/site/about/guidelines.xhtml

(25) Medical Research Institute of New Zealand. *Taking Charge After Stroke (TaCAS)*. Available from: https://mrinz.ac.nz/programmes/stroke/ [Accessed 07 oct 2019].

(26) Hibbard JH, Stockard J, Mahoney ER, Tusler M. Development of the Patient Activation Measure (PAM): Conceptualizing and Measuring Activation in Patients and Consumers. *Health Services Research*. 2004; 39 (4p1): 1005-1026. Available from: doi: 10.1111/j.1475-6773.2004.00269.x Available from: https://onlinelibrary.wiley.com/doi/abs/10.1111/j.1475-6773.2004.00269.x .

(27) Roberts NJ, Kidd L, Dougall N, Patel IS, McNarry S, Nixon C. Measuring patient activation: The utility of the Patient Activation Measure within a UK context—Results from four exemplar studies and potential future applications. *Patient Education and Counseling*. 2016; 99 (10): 1739-1746. Available from: doi: 10.1016/j.pec.2016.05.006 Available from: https://www.clinicalkey.es/playcontent/1-s2.0-S0738399116301938 .

(28) Sahlgrenska Academy Institute of Neuroscience and Physiology, University of Gothenburg. *Fugl-Meyer*. Available from: https://neurophys.gu.se/english/departments/clinical_neuroscience_and_rehabilitation/rehabilitation-medicine/fugl-meyer [Accessed 04 Oct 2019].

(29) Pamela W Duncan, Martha Propst, Steven G Nelson. Reliability of the Fugl-Meyer Assessment of Sensorimotor Recovery Following Cerebrovascular Accident. *Physical Therapy*. 1983; 63 (10): 1606-1610. Available from: doi: 10.1093/ptj/63.10.1606 Available from: http://ptjournal.apta.org/content/63/10/1606.abstract .

(30) Uswatte G, Taub E, Morris D, Vignolo M, McCulloch K. Reliability and Validity of the Upper-Extremity Motor Activity Log-14 for Measuring Real-World Arm Use. *Stroke*. 2005; 36 (11): 2493-2496. Available from: doi: 10.1161/01.STR.0000185928.90848.2e Available from: http://stroke.ahajournals.org/cgi/content/abstract/36/11/2493 .

(31) Quinn TJ, Dawson J, Walters MR, Lees KR. Exploring the Reliability of the Modified Rankin Scale. *Stroke*. 2009; 40 (3): 762-766. Available from: doi: 10.1161/STROKEAHA.108.522516 Available from: http://stroke.ahajournals.org/cgi/content/abstract/40/3/762 .

(32) Jensen MP, Karoly P, Braver S. The measurement of clinical pain intensity: a comparison of six methods. *Pain*. 1986; 27 (1): 117-126. Available from: doi: 10.1016/0304-3959(86)90228-9 Available from: https://www.sciencedirect.com/science/article/pii/0304395986902289 .

(33) Nasreddine ZS, Phillips NA, Bédirian V, Charbonneau S, Whitehead V, Collin I, et al. The Montreal Cognitive Assessment, MoCA: A Brief Screening Tool For Mild Cognitive Impairment. *Journal of the American Geriatrics Society*. 2005; 53 (4): 695-699. Available from: doi: 10.1111/j.1532-5415.2005.53221.x Available from: https://onlinelibrary.wiley.com/doi/abs/10.1111/j.1532-5415.2005.53221.x .

(34) *MoCA Test - Remote MoCA Testing*. Available from: https://www.mocatest.org/remote-moca-testing/ [Accessed 09 September 2021].

(35) Fullerton KJ, McSherry D, Stout RW. Albert's test: a neglected test of perceptual neglect. *Lancet (London, England)*. 1986; 1 (8478): 430. Available from: https://www.ncbi.nlm.nih.gov/pubmed/2868349 .

(36) EuroQol - a new facility for the measurement of health-related quality of life. *Health policy*. 1990; 16 (3): 199-208. Available from: doi: 10.1016/0168-8510(90)90421-9 Available from: https://www.sciencedirect.com/science/article/pii/0168851090904219 .

(37) SUS: A 'Quick and Dirty' Usability Scale. *Usability Evaluation In Industry*. CRC Press; 1996. pp. 207-212.

(38) Billingham SAM, Whitehead AL, Julious SA. An audit of sample sizes for pilot and feasibility trials being undertaken in the United Kingdom registered in the United Kingdom Clinical Research Network database. *BMC medical research methodology*. 2013; 13 (1): 104. Available from: doi: 10.1186/1471-2288-13-104 Available from: https://www.ncbi.nlm.nih.gov/pubmed/23961782 .

For peer review only - http://bmjopen.bmj.com/site/about/guidelines.xhtml

(39) National Institute for Health Research. *About INVOLVE*. Available from: https://www.invo.org.uk/about-involve/ [Accessed 04 Oct 2019].

(40) Scheff SW. Nonparametric Statistics. *Fundamental Statistical Principles for the Neurobiologist*. Elsevier Inc; 2016. pp. 157-182.

(41) Lang CE, Edwards DF, Birkenmeier RL, Dromerick AW. Estimating Minimal Clinically Important Differences of Upper-Extremity Measures Early After Stroke. *Archives of physical medicine and rehabilitation*. 2008; 89 (9): 1693-1700. Available from: doi: 10.1016/j.apmr.2008.02.022 Available from: https://search.datacite.org/works/10.1016/j.apmr.2008.02.022 .

(42) Burridge JH, Lee ACW, Turk R, Stokes M, Whitall J, Vaidyanathan R, et al. Telehealth, Wearable Sensors, and the Internet: Will They Improve Stroke Outcomes Through Increased Intensity of Therapy, Motivation, and Adherence to Rehabilitation Programs? Journal of neurologic physical therapy. 2017; 41 Suppl 3 Supplement, IV STEP Special Issue S32-S38. Available from: doi: 10.1097/NPT.0000000000000183 Available from: http://ovidsp.ovid.com/ovidweb.cgi?T=JS&NEWS=n&CSC=Y&PAGE=fulltext&D=ovft&AN=01253086-201707001-00006 .

FIGURE LEGEND

Figure 1 - CONSORT flow diagram

Figure 2 - Average days recording per week (min, max)

Figure 3 - Average daily data views on phone and watch

Figure 4 - Weekly activity, target, and number of days the target was reached

Figure 5 - Overall messages sent / opened (daily average)

For peer review only

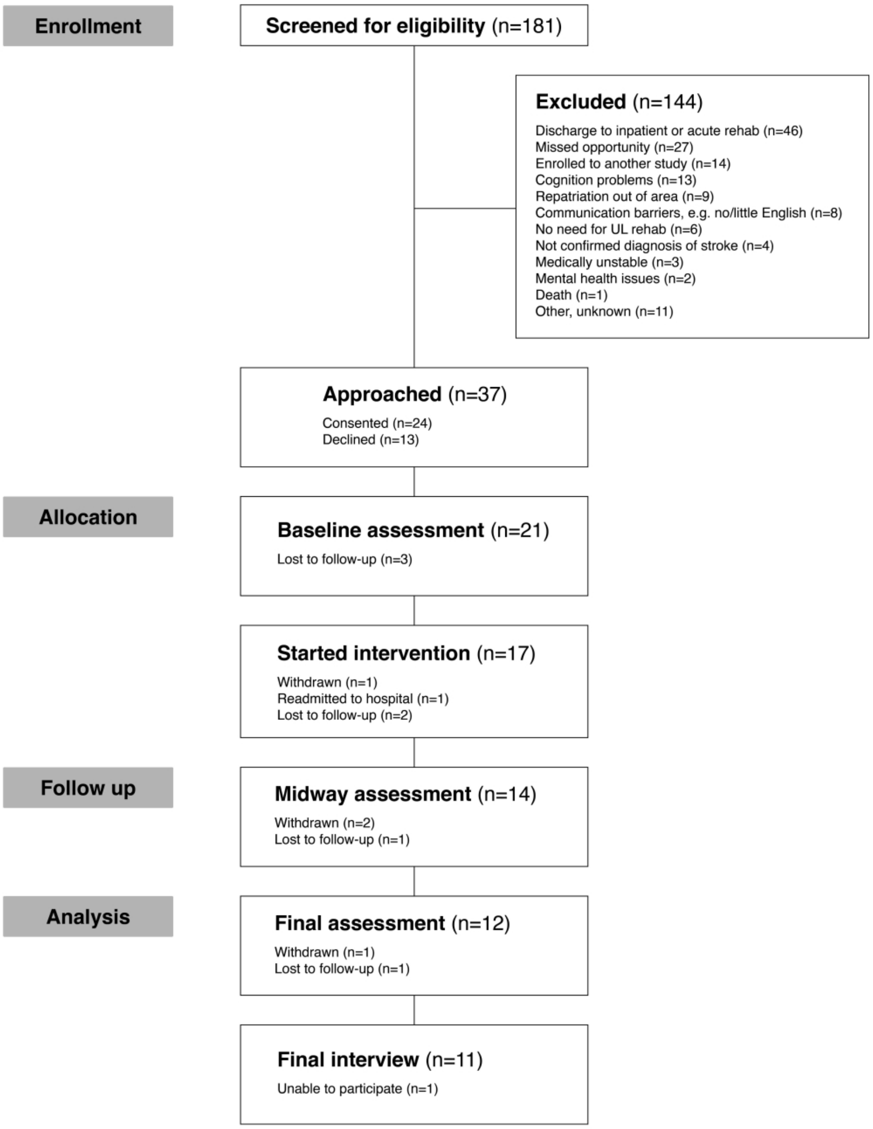

Figure 1 - CONSORT flow diagram

1609x2063mm (72 x 72 DPI)

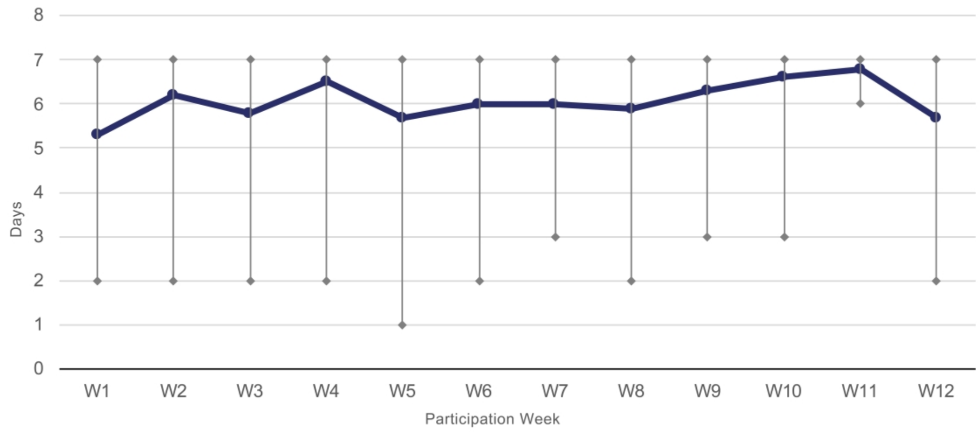

Figure 2 - Average days recording per week (min, max)

116x51mm (1200 x 1200 DPI)

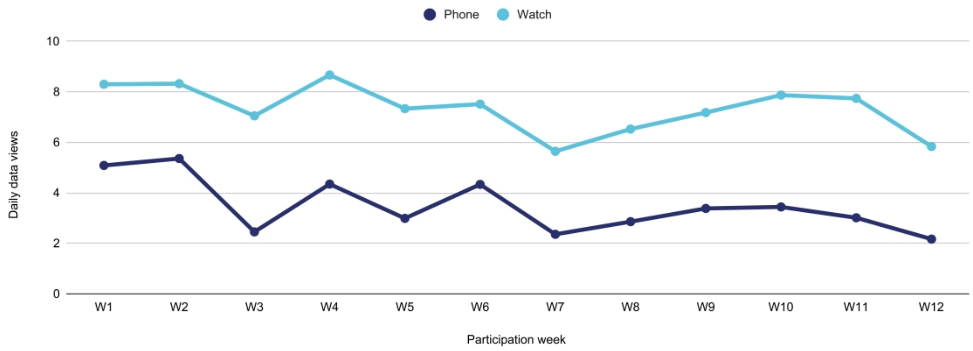

Figure 3 - Average daily data views on phone and watch

173x65mm (1200 x 1200 DPI)

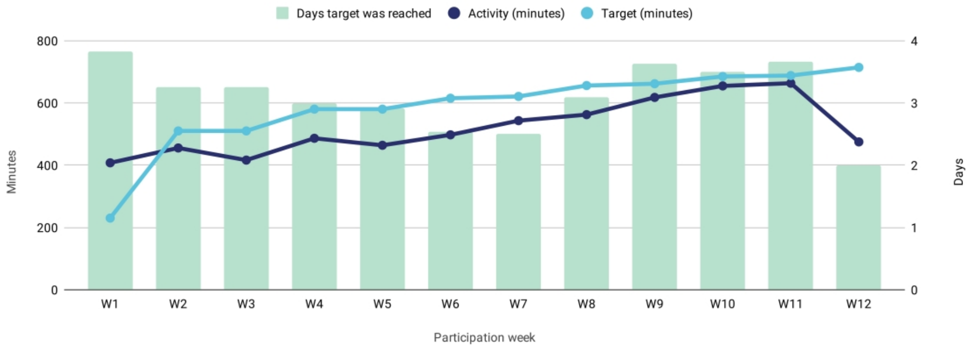

Figure 4 - Weekly activity, target, and number of days the target was reached

173x65mm (1200 x 1200 DPI)

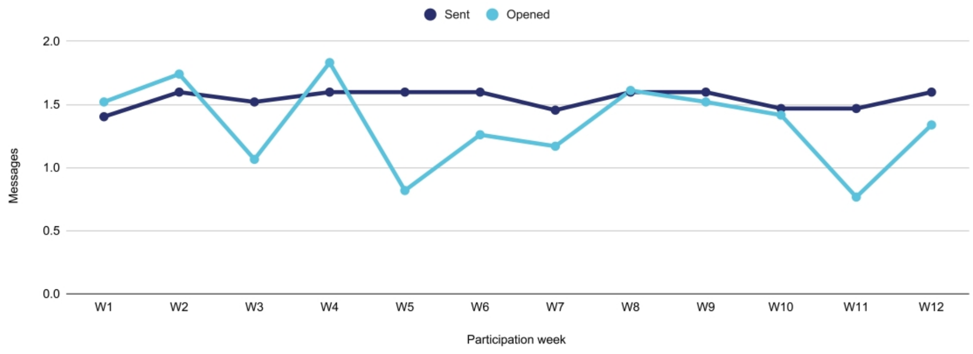

Figure 5 - Overall messages sent / opened (daily average)

173x65mm (1200 x 1200 DPI)

Supplementary file 1 - Phase 1 and Phase 2 coaching structures

Coaching delivery - Study phase 1

Week	Session	Coaching phase	Coaching guidelines
W1	Baseline assessment	*No coaching*	
W2	Coaching session 1	Onboarding	Introduction to OnTrack / purpose, value, practical issues
W3	Coaching session 2	Problem solving	Encouraging people to discuss areas where they struggle, then facilitate coming up with ideas and strategies around problems faced during daily life situations.
W4			
W5	Coaching session 3	Self-discovery	Finding out new ways of doing things, trying out different activities, so that any attempt to try and find a new way around something is seen as a positive.
W6			
W7	Coaching session 4	Goal setting	Working with participants to identify, plan and execute steps towards a goal regardless of size and ambition. The aim is to help participants learn and get comfortable with this process under their terms.<br><br>Outcome measure collection.
W8	Halfway assessment		
W9			
W10	Coaching session 5	Reflection	Looking back at achievements and failures to understand what worked and what didn't in order to adjust if necessary and look forward to where they want to be.
W11			
W12	Coaching session 6	Signposting	Using goals and aspirations to help participants create links that encourage social participation, community integration and/or peer support.
W13			
W14	Final assessment	*No coaching*	

Coaching delivery - Study phase 2

Week	Session	Coaching phase	Coaching guidelines
W1	Baseline assessment	*No coaching*	Initial contact; conversation about participant's background and present situation.
W2	Coaching session 1	Introduction	Focus on creating a habit of using OnTrack and charging devices. Setting the scene for coaching.
W3	Coaching session 2	OnTrack coaching	In-depth conversations about participants' aspirations and what's important to them.<br><br>Discussion to assess participants' understanding of stroke and rehab mechanisms, identify topics of curiosity, signpost as necessary.<br><br>Use of self-management principles (Problem Solving, Self-
W4			
W5	Coaching session 3		

For peer review only

W6			discovery, Goal Setting, and Reflection) and rehab guidelines to focus conversations towards reaching an agreed activity plan (however small).<br><br>Make connections between activity performed and data recorded.
W7	Halfway assessment		
W8			
W9	Coaching session 4		
W10			
W11	Coaching session 5		
W12			
W13	Coaching session 6	Signposting	Signpost to relevant resources that can be accessed by participants going forward.
W14	Final assessment	*No coaching*	Closure; last query to ensure participants feel confident to continue their rehab on their own.

For peer review only

Reporting checklist for quality improvement in health care.

Based on the SQUIRE guidelines.

Instructions to authors

Complete this checklist by entering the page numbers from your manuscript where readers will find each of the items listed below.

Your article may not currently address all the items on the checklist. Please modify your text to include the missing information. If you are certain that an item does not apply, please write "n/a" and provide a short explanation.

Upload your completed checklist as an extra file when you submit to a journal.

In your methods section, say that you used the SQUIREreporting guidelines, and cite them as:

Ogrinc G, Davies L, Goodman D, Batalden P, Davidoff F, Stevens D. SQUIRE 2.0 (Standards for QUality Improvement Reporting Excellence): revised publication guidelines from a detailed consensus process

		Reporting Item	Page Number
**Title**			
	#1	Indicate that the manuscript concerns an initiative to improve healthcare (broadly defined to include the quality, safety, effectiveness, patientcenteredness, timeliness, cost, efficiency, and equity of healthcare)	1
**Abstract**			
	#02a	Provide adequate information to aid in searching and indexing	1
	#02b	Summarize all key information from various sections of the text using the abstract format of the intended publication or a structured summary such as: background, local problem, methods, interventions, results, conclusions	1
**Introduction**			
Problem description	#3	Nature and significance of the local problem	3

For peer review only

Available knowledge	#4	Summary of what is currently known about the problem, including relevant previous studies	3
Rationale	#5	Informal or formal frameworks, models, concepts, and / or theories used to explain the problem, any reasons or assumptions that were used to develop the intervention(s), and reasons why the intervention(s) was expected to work	3
Specific aims	#6	Purpose of the project and of this report	3
**Methods**			
Context	#7	Contextual elements considered important at the outset of introducing the intervention(s)	4
Intervention(s)	#08a	Description of the intervention(s) in sufficient detail that others could reproduce it	4
Intervention(s)	#08b	Specifics of the team involved in the work	4
Study of the Intervention(s)	#09a	Approach chosen for assessing the impact of the intervention(s)	4
Study of the Intervention(s)	#09b	Approach used to establish whether the observed outcomes were due to the intervention(s)	4
Measures	#10a	Measures chosen for studying processes and outcomes of the intervention(s), including rationale for choosing them, their operational definitions, and their validity and reliability	6
Measures	#10b	Description of the approach to the ongoing assessment of contextual elements that contributed to the success, failure, efficiency, and cost	6
Measures	#10c	Methods employed for assessing completeness and accuracy of data	6
Analysis	#11a	Qualitative and quantitative methods used to draw inferences from the data	7
Analysis	#11b	Methods for understanding variation within the data, including the effects of time as a variable	7
Ethical considerations	#12	Ethical aspects of implementing and studying the intervention(s) and how they were addressed, including, but	2

For peer review only

		not limited to, formal ethics review and potential conflict(s) of interest	
**Results**			
	#13a	Initial steps of the intervention(s) and their evolution over time (e.g., time-line diagram, flow chart, or table), including modifications made to the intervention during the project	7
	#13b	Details of the process measures and outcome	8
	#13c	Contextual elements that interacted with the intervention(s)	8
	#13d	Observed associations between outcomes, interventions, and relevant contextual elements	10
	#13e	Unintended consequences such as unexpected benefits, problems, failures, or costs associated with the intervention(s).	9
	#13f	Details about missing data	9
**Discussion**			
Summary	#14a	Key findings, including relevance to the rationale and specific aims	16
Summary	#14b	Particular strengths of the project	16
Interpretation	#15a	Nature of the association between the intervention(s) and the outcomes	16
Interpretation	#15b	Comparison of results with findings from other publications	16
Interpretation	#15c	Impact of the project on people and systems	16
Interpretation	#15d	Reasons for any differences between observed and anticipated outcomes, including the influence of context	16
Interpretation	#15e	Costs and strategic trade-offs, including opportunity costs	n/a clinical feasibility and acceptability study
Limitations	#16a	Limits to the generalizability of the work	16
Limitations	#16b	Factors that might have limited internal validity such as confounding, bias, or imprecision in the design, methods, measurement, or analysis	16

For peer review only

Limitations	#16c	Efforts made to minimize and adjust for limitations	n/a clinical feasibility and acceptability study
Conclusion	#17a	Usefulness of the work	16
Conclusion	#17b	Sustainability	n/a clinical feasibility and acceptability study
Conclusion	#17c	Potential for spread to other contexts	n/a clinical feasibility and acceptability study
Conclusion	#17d	Implications for practice and for further study in the field	16
Conclusion	#17e	Suggested next steps	16
**Other information**			
Funding	#18	Sources of funding that supported this work. Role, if any, of the funding organization in the design, implementation, interpretation, and reporting	17

The SQUIRE 2.0 checklist is distributed under the terms of the Creative Commons Attribution License CC BY-NC 4.0. This checklist was completed on 10. February 2022 using https://www.goodreports.org/, a tool made by the EQUATOR Network in collaboration with Penelope.ai

For peer review only
